# Novel QTL for Lateral Root Density and Length Improve Phosphorus Uptake in Rice (*Oryza sativa* L.)

**DOI:** 10.1186/s12284-023-00654-z

**Published:** 2023-08-24

**Authors:** Lam Thi Dinh, Yoshiaki Ueda, Daniel Gonzalez, Juan Pariasca Tanaka, Hideki Takanashi, Matthias Wissuwa

**Affiliations:** 1 Crop, Livestock and Environment Division, Japan International Research Center for Agricultural Sciences (JIRCAS), 1-1 Ohwashi, Tsukuba, Ibaraki 305-8686 Japan; 2 Present Address: Department of Applied Biology and Food Sciences, Faculty of Agriculture and Life Science, Hirosaki University, Hirosaki, Aomori 036-8561 Japan; 3 Graduate School of Agricultural and Life Sciences, The University of Tokyo, Tokyo, Japan; 4 PhenoRob Cluster and Institute of Crop Science and Resource Conservation (INRES), University of Bonn, Bonn, Germany; 5 Department of Plant protection, Institute of Agricultural Science for Southern Vietnam (IAS), Ho Chi Minh City, Vietnam

**Keywords:** L-type lateral roots, S-type lateral roots, Crown root, P uptake simulation, Root system architecture

## Abstract

The rice root system consists of two types of lateral roots, indeterminate larger L-types capable of further branching, and determinate, short, unbranched S-types. L-type laterals correspond to the typical lateral roots of cereals whereas S-type laterals are unique to rice. Both types contribute to nutrient and water uptake and genotypic variation for density and length of these laterals could be exploited in rice improvement to enhance adaptations to nutrient and water-limited environments. Our objectives were to determine how best to screen for lateral root density and length and to identify markers linked to genotypic variation for these traits. Using different growing media showed that screening in nutrient solution exposed genotypic variation for S-type and L-type density, but only the lateral roots of soil-grown plants varied for their lengths. A QTL mapping population developed from parents contrasting for lateral root traits was grown in a low-P field, roots were sampled, scanned and density and length of lateral roots measured. One QTL each was detected for L-type density (LDC), S-type density on crown root (SDC), S-type density on L-type (SDL), S-type length on L-type (SLL), and crown root number (RNO). The QTL for LDC on chromosome 5 had a major effect, accounting for 46% of the phenotypic variation. This strong positive effect was confirmed in additional field experiments, showing that lines with the donor parent allele at *qLDC5* had 50% higher LDC. Investigating the contribution of lateral root traits to P uptake using stepwise regressions indicated LDC and RNO were most influential, followed by SDL. Simulating effects of root trait differences conferred by the main QTL in a P uptake model confirmed that *qLDC5* was most effective in improving P uptake followed by *qRNO9* for RNO and *qSDL9* for S-type lateral density on L-type laterals. Pyramiding *qLDC5* with *qRNO9* and *qSDL9* would be possible given that trade-offs between traits were not detected. Phenotypic selection for the RNO trait during variety development would be feasible, however, the costs of doing so reliably for lateral root density traits is prohibitive and markers identified here therefore provide the first opportunity to incorporate such traits into a breeding program.

**Supplementary Information:**

The online version contains supplementary material available at 10.1186/s12284-023-00654-z.

## Introduction

Rice (*Oryza sativa*) is the most important staple food for the world’s human population, providing 20% of the global dietary energy supply, while wheat supplies 19% and maize 5% (FAO 2004). Rice production is currently gaining importance in Africa. Farmers in about 40 out of 54 African countries are growing rice, making cultivation of this crop a principal source of food and income for more than 35 million smallholder rice farmers in Africa (https://www.africarice.org/, https://www.fao.org/africa/news/detail-news/en/c/1612346/). However, rice consumption is increasing even more rapidly, particularly in urban areas of Africa, and the current production is going to meet only two-thirds of the projected consumption of 34.9 million tons of rice by 2025. The combination of low production inputs and unfavorable production environments are considered to be the main reasons for the relatively low productivity in Sub-Saharan Africa (SSA) (Saito et al. 2019), with deficiency of phosphorus (P) being one of the major constraints. Breeding rice varieties with improved P-efficiency could make an important contribution to boosting rice productivity where soil-P availability is low and low-income farmers are unable to purchase P fertilizers. Genotypic differences in P uptake from low-P soils exist in rice and are mainly caused by genotypic differences in root size, and to a lesser extent in root efficiency (Mori et al. 2016; Wissuwa et al. 2020). Rice accession DJ123 of Bangladeshi origin has been identified as producing relatively high grain yields on low-P soils in West-Africa, whereas the local recommended variety Nerica4 was sensitive to P deficiency (Vandamme et al. 2016). Subsequent studies conducted on a highly P-fixing soil in Japan have shown that DJ123 combined higher internal P utilization efficiency with faster root development and higher P acquisition efficiency (P uptake per unit root size) compared to Nerica4 (Wissuwa et al. 2015, 2020). DJ123 has therefore been used as a donor to improved P efficiency in Nerica4 and breeding populations derived from the DJ123 × Nerica4 cross have been evaluated on a P deficient soil in Madagascar where it was confirmed that DJ123 had more rapid root development (Ranaivo et al. 2022).

**Fig. 1:**
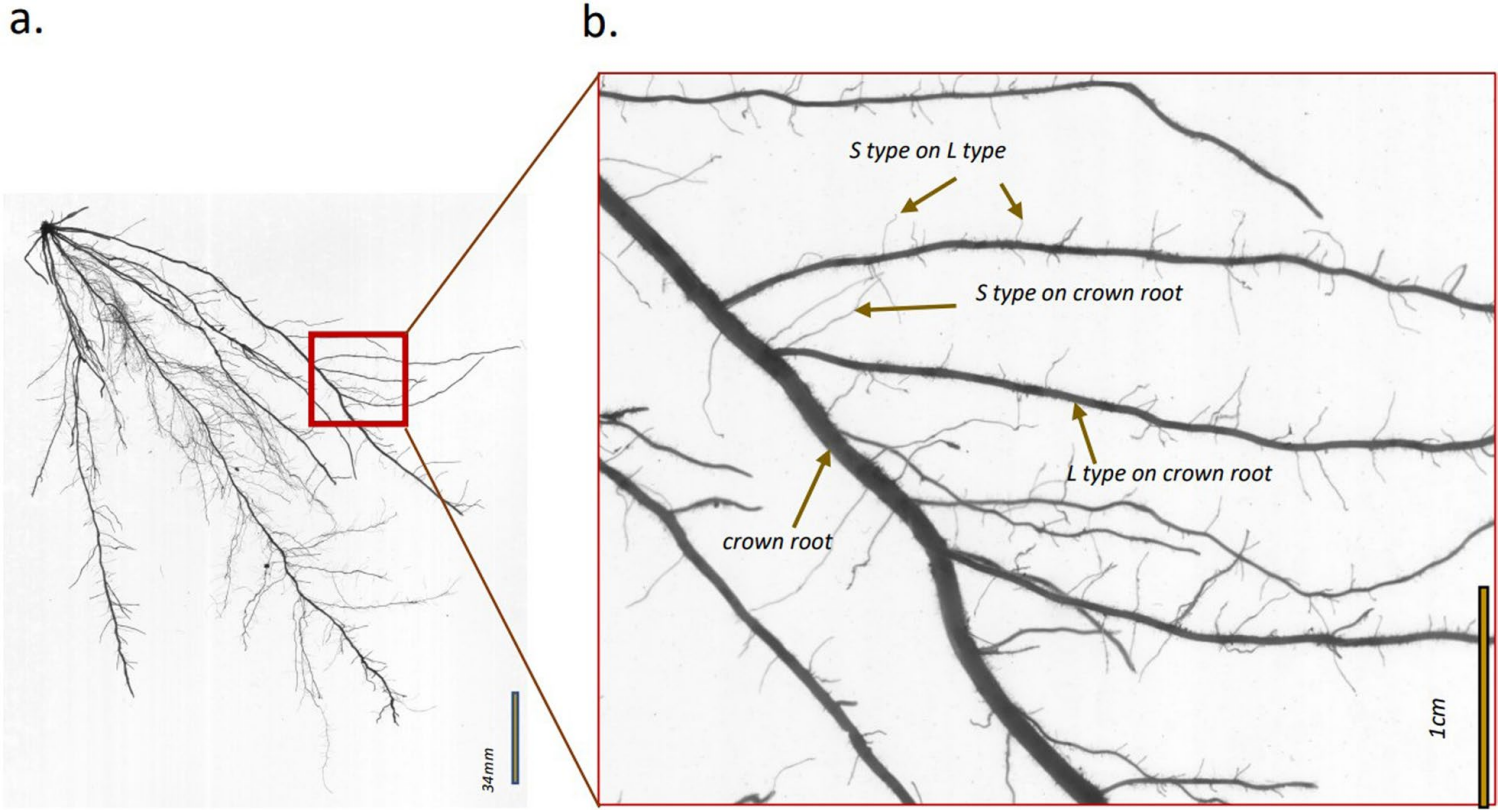
Scan of a root system excavated 41 DAS from a low-P field experiment (**a**) and classification of L-type and S-type lateral roots in relation to their parent root (**b**). The scan was obtained using an Epson Perfection V700 photo dual-lens scanner with top lighting, with the following settings: 600 dpi, 16-bit grayscale, positive film

The rice root system is comprised of four main root classes: main root axes are formed by the primary root and subsequent nodal roots (also called crown roots) and both classes give rise to two classes of lateral roots (Rebouillat et al. 2009) (Fig. 1). The larger L-type lateral roots contain several layers of cortex cells and typically have secondary or even tertiary branches while the small S-type lateral roots contain only one layer of cortical cells, are unbranched and short with a maximum length of around 1 cm and a diameter around 50 µm (Wissuwa et al. 2020). S-type laterals develop on both crown roots and L-type laterals. An order of magnitude finer than these S-type laterals are root hairs that develop on all root classes in rice, including the already fine S-type laterals (Nestler et al. 2016). It becomes evident that rice has a particularly high proportion of very fine root structures exploring the soil for resources like water and nutrients, which should be especially beneficial for poorly mobile nutrients like P.

Attempts have been made to estimate the contribution of each root class to plant P uptake and this has predominantly relied on model simulations (De Bauw et al. 2020; Gonzalez et al. 2021; Kuppe et al. 2022). In a study varying soil water and P supply, De Bauw et al. (2020) concluded that both lateral root classes contribute more than crown roots to P uptake. This was in part due to the assumption that root tips growing into yet unexplored soil would have access to more P; total tip number being several orders of magnitude higher for lateral compared to crown roots. Furthermore, L-type laterals became increasingly important for water and P uptake in drying soil. Using a different 3-D rice root model that included root hairs on all four root classes attributed a larger role in P uptake to crown roots, which was due to their large diameter and root surface area coupled with a dense cover by root hairs (Gonzalez et al. 2021). Their model sensitivity analysis suggested that increasing density and length of L-type lateral roots would have most pronounced benefits for P uptake. When root-induced P solubilization processes in the rhizosphere were considered in a model in addition to root hairs, the relative contribution to P uptake of S-type lateral roots (and their hairs) increased because they were able to take up P solubilized by their parent root at a distance too far for P to diffuse back to the parent root (Kuppe et al. 2022).

A common feature of these recent modeling studies was to highlight the importance of lateral roots for P (and water) uptake. That genotypic differences in P uptake were largely driven by differences in root size is well established (Mori et al. 2016; Wissuwa et al. 2020). However, these recent studies highlight that some root classes may be more influential than others and may thus be targeted for selection in rice breeding (Gonzalez et al. 2021). Genotypic differences in crown root number have been reported and associated QTL identified (Hemamalini et al. 2000; Ranaivo et al. 2022). Studies on lateral root traits are scarce, however, Niones et al. (2015) identified a QTL for L-type lateral root density on chromosome 8. The same study reported a QTL for total lateral root density on chromosome 12 whereas Wang et al. (2018) described a QTL for total root tip number on chromosome 11. To our knowledge loci for S-type lateral root densities or length have not been identified, nor has a distinction been made between lateral root branching densities on different parent root classes (e.g. S-type density on crown roots *versus* on L-type lateral roots). For the branching density of root hairs on parent roots, Nestler et al. (2016) detected strong effects of the screening medium, with dense branching being largely restricted to the crown root when plants were grown in nutrient solution, whereas root hairs developed on all lateral roots in soil.

Lateral root traits are potentially important for P and water uptake but little is known about the extent of genotypic variation for these traits, about possible genetic factors underlying naturally occurring variation within the rice gene pool, and to what extent genotypic variation can be detected by different screening methods. Our objectives were to address these knowledge gaps by (1) assessing genotypic differences for lateral root traits in upland rice genotypes DJ123, Nerica4, and NDJ188; (2) identifying a suitable screening method that would reliably exhibit genotypic differences; (3) identifying QTL controlling these traits utilizing a QTL mapping population derived from P efficient donor DJ123 and inefficient recurrent parent Nerica4; and (4) examining to what extent detected QTL contribute to differences in P uptake under P limiting conditions.

## Materials and Methods

### Plant Material

An initial breeding population targeting P-deficient upland environments in SSA had been developed at the Africa Rice Center from a cross of P-efficient genebank accession DJ123 with P inefficient upland variety Nerica4 (Ranaivo et al. 2022). DJ123 belongs to the *aus* subpopulation of rice whereas Nerica4 had been developed from an interspecific cross between CG14, an *Oryza glaberrima* donor, and recurrent parent WAB56-104 belonging to the *tropical japonica* subpopulation of *Oryza sativa*. The initial DJ123 × Nerica4 population was provided by the Africa Rice Center to JIRCAS and its partner FOFIFA in Madagascar. Among them, line NDJ188 was found to have good field performance under low-input conditions in Madagascar (Ranaivo et al. 2022) and was back-crossed to Nerica4 to develop a BC1 QTL mapping population comprising 201 lines. These were genotyped and phenotyped in the derived BC1F5 generation with 98 selected lines selected based on high homozygosity in the BC1F3 generation. The detected QTL were confirmed in selected BC1F6 lines that contrast in detected loci.

### Variation in Lateral Root Traits Between Parental Genotypes (Experiment 1)

Lateral root length and density were characterized in parental genotypes DJ123, Nerica4, and NDJ188 in a nutrient solution, rhizobox, and field experiments. Plants were grown with Yoshida nutrient solution (Yoshida et al. 1972) in which the standard P concentration of 320 µM was reduced to 2 µM to evaluate lateral root traits under P deficiency. The experiment was conducted in 44-L containers in a randomized complete block design with 4 replications. Seeds were sterilized (1% w/v NaClO for 5 min) and germinated in petri dishes at 30 °C, in dark for 2 days. Germinated seeds were then transferred onto a mesh floating in trays containing 8 L of solution (100 µM Ca, CaCl_2_; 10 µM Fe, Fe-EDTA; pH 5.8), and nursed in the glasshouse. At 10 days after sowing (DAS), seedlings of uniform size were selected for each genotype and transferred to 44 L containers containing the Yoshida nutrient solution with 2 µM P. The solution pH was adjusted to 5.7 every 2–3 days and a new dose of P was added twice weekly. The entire solution was exchanged weekly. Plants were harvested 35 DAS.

The rhizobox experiment used a field soil collected at the JIRCAS experimental farm in Tsukuba, Japan. The soil is a highly P fixing Andosol with low-P availability of 7 mg kg^−1^ (Bray II). It is from the same field described in Ranaivo et al. (2022) and used in field experiment 2 described below. Air-dried soil was used to fill the Plexiglas rhizoboxes of dimensions 30 × 30 × 2 cm (height × width × depth for inner dimensions). A single plant was grown in each rhizobox, the experiment was conducted as a completely randomized block design with 4 replications and plants were sampled at 35 DAS. Roots were stored with water in bags at 4 °C until root scans were obtained. Parental lines were further characterized in a fourth experiment conducted between March and June 2021 at the JIRCAS experimental farm in Ishigaki Island, Okinawa, Japan. A field that had never received P fertilizer was used as a low P plot, while a field with conventional fertilizer application was used as a control plot. Seeds of each genotype were sown in a single row of 1.1 m in length with a spacing of 20 cm between rows. After emergence rows were thinned to 13 plants per row. Roots of three plants per genotype per replication (in total 3 replications) were excavated at 60 DAS, gently washed then stored in zipped freezer bags with a small amount of water until root scanning.

### Phenotyping Lateral Root Traits in the QTL Mapping Population (Experiments 2 and 3)

Lines deriving from the QTL mapping population (n = 98) were grown between June-July 2021 along with their parents DJ123, Nerica4, NDJ188 at the JIRCAS experimental field station in Tsukuba, Japan. Parents and 98 BC1F5 lines of the mapping population were sown in a randomized complete block design (RCBD) with two replications on 2nd June 2021. Each entry was sown in a single row of 1.1 m in length with a spacing of 15 cm between rows. After emergence rows were thinned to 13 plants per row on 21st June 2021. The field was occasionally supplied with water to prevent drought stress and weeds were manually removed. Three plants per line per replicate plot were sampled at 41 DAS. Plants were dug out to a depth of 20 cm using a spade and soil was washed off with water. The number of crown roots per plant (RNO) was counted, after which roots were placed in bags containing enough water to prevent drying and stored at 4 °C until root scans were obtained. Shoots were oven-dried for 3 days at 70 °C and weighed. A subsample of 0.2 g was used for acid digestion in a 3:1 mixture of HNO_3_ and HClO_4_ and subjected to determination of tissue P concentrations using the molybdenum blue method as previously described (Prodhan et al. 2022).

To confirm the effects of detected QTL, lines contrasting for the main QTL were selected from the BC1F5 population (n = 32) and grown in a 3rd field experiment during June-July 2022. Sampling of roots and shoots was done at 40 DAS using the same procedures as described for experiment 2.

### Root Scanning and Analysis

Roots were spread out in a 20 × 25 cm Perspex tray filled with water to a depth of 0.5 cm and scanned using an Epson Perfection V700 photo dual-lens scanner with top lighting, with the following settings: 600 dpi, 16-bit grayscale, positive film. Obtained images were analyzed by ImageJ software (Schneider et al. 2012). The L-type lateral root density on crown roots (LDC) was estimated by counting the number of L-type lateral roots per 2 cm long crown root section. The value was obtained from a total of 10 sections per plant selected from the branched parts of roots, avoiding unbranched root tip regions and fast-growing crown roots known for their reduced lateral branching (Gonzalez et al. 2021). Similar measurements were made for the S-type lateral root density on crown roots (SDC) and S-type lateral root density on L-type lateral roots (SDL), each from ten sections of 2 cm length. The data was converted into root density per 1 cm unit for further analysis. The L-type and S-type lateral root length was measured to obtain estimates for L-type lateral root length on crown root (LLC), S-type lateral root length on crown root (SLC), and S-type lateral root length on L-type lateral root (SLL). Up to 20 SLC, SLL, and 10 longest L-type roots per scan were measured using the ImageJ freehand line tool with the scale set for Distance in pixels: 756; Known distance: 340 based on an object of known length; Pixel aspect ratio: 1.0, and unit of length was mm to estimate a single L-type lateral root length. Twenty typical S-type lateral roots on crown roots and on L-type lateral roots per scan were measured to estimate a single S-type lateral root length. S-type lateral roots close to the root tip of the crown root or L-type lateral root were classified as young roots and were not measured. The setting was the same as for the L-type lateral root length described above.

### SNP Identification and Genotyping

The mapping population was genotyped by restriction site-associated DNA sequencing (RAD-Seq) (Baird et al. 2008) and SNP markers were developed essentially as reported previously (Ranaivo et al. 2022). Briefly, a RAD-Seq library was prepared by digesting genomic DNA with BglII and MseI (Kobayashi et al. 2017). The resultant library was sequenced with the Hiseq-X instrument (Illumina). The raw reads were checked for quality by FastQC software (ver 0.11.9) (https://www.bioinformatics.babraham.ac.uk/projects/fastqc/) and trimmed by trimmomatic software (ver 0.38) (Bolger et al. 2014) using the following settings: LEADING:3 TRAILING:3 SLIDINGWINDOW:4:15 MINLEN:100. These sequences were aligned to the publicly available genome sequence assembly of a *tropical japonica* cultivar Azucena (NCBI BioProject PRJNA424001) using the bwa software (ver 0.7.17) (Li and Durbin 2009). Aligned reads were ordered, indexed and converted to BAM format with samtools (ver 1.9) (Li et al. 2009). Variants were extracted using bcftools software (ver 1.9) (Li et al. 2009) and the extracted SNPs were filtered by vcftools software (ver 0.1.16) (Danecek et al. 2011) using the following settings: —min-meanDP 10—max-meanDP 100—max-missing 0.95—minQ 20—min-alleles 2—max-alleles 2. Monomorphic markers, indel markers and SNPs within 20 bp of detected indel sites were further removed. A heterozygous allele was defined only when > 5 reads from each parent supported the genotype. Missing alleles were imputed by the *k*-nearest neighbors imputation method using TASSEL 5 software (Bradbury et al. 2007; Money et al. 2015). Monomorphic and indel markers were further removed. To generate genotype data for QTL mapping, markers not polymorphic between Nerica4 and DJ123 were removed as were markers with minor allele frequency < 0.1 and heterozygosity > 0.2. In the final step markers within a 200 kb distance of an adjacent marker were removed, leaving a set of 565 SNP markers.

### Genetic Linkage Map Construction and QTL Analysis

SNP markers were converted to ABH-format, where ‘A’ denotes the donor allele (DJ123), ‘B’ the recipient parent allele (Nerica4), and ‘H’ the heterozygous state. Software packages Rqtl and LinkageMapView (Broman et al. 2003; Ouellette et al. 2018) were used to calculate genetic distances and to build the linkage map for 565 SNP markers. The marker order was initially based on their physical map positions, but the final order was determined after applying the ripple function in Rqtl. Genetic distances were estimated in cM using the Kosambi option.

The linkage map was imported into the software QGENE (Joehanes and Nelson 2008) for QTL analysis. Median phenotypic values of 10 counts for lateral root density traits (LDC, SDC, SDL) and 20 counts for lateral root length traits (LLC, SLC, SLL) were used as phenotypic data. Putative QTL were detected by Composite Interval Mapping, in which the most influential markers based on an F-to-enter of 8.0 were used as cofactors in the analysis. The significance threshold for each trait was determined using a permutation test with 1000 iterations at a significance level of 5%.

For the confirmation of detected QTL in the BC1F6, 32 contrasting lines were selected and the effect of substituting Nerica4 (BB) alleles by DJ123 (AA) alleles was estimated based on mean phenotypic values of respective classes using Tukey HSD All-Pairwise Comparisons Test.

### Broad Sense Heritability

The variance components and broad sense heritability for seven root traits were estimated based on mean values of BC1F5 population obtained from experiment 2, and calculated using the “variability” package in R (Popat et al. 2020).

### Modeling P Uptake for Allelic Effects at QTL *qLDC5*, *qRNO9 *and *qSDL9*

In order to simulate effects on root growth and P uptake of allelic differences at QTL *qLDC5, qRNO9*, and *qSDL9*, the OpenSimRoot rice model (Gonzalez et al. 2021) currently found in the OpenSimRoot gitlab (https://gitlab.com/rootmodels/OpenSimRoot) was used. The base model had been built for DJ123 and was run without modifications. To simulate effects of replacing DJ123 alleles by Nerica4 alleles, model parameters were adjusted as follows: crown root number was reduced by 10.1%, and Inter branching distance (IBD) between L-type lateral roots or S-type laterals on L-type laterals was increased by 33.8% and 16.8%, respectively. The model was run to 30 days with a 0.2 day timestep calculation. Each run was made with a stochastic sample of 1000 in order to minimize stochastic effects on results.

A stepwise linear regression analysis was conducted in Statistix 10 (https://www.statistix.com) to determine the contribution of all studied root traits to root system size and subsequent P uptake. Further, we calculated a simple Root Index for each line by multiplying its values for RNO, LDC and SDL to compare with modeling and stepwise linear regression results.

## Results

### Effect of Screening Environment and Genotype on Lateral Root Traits

Parental genotypes were screened in nutrient solution and two soil-based environments (rhizobox and field) and distinct differences between nutrient solution and soil were observed for all six root traits (Table 1). Roots grown in nutrient solution had much higher S-type lateral root densities, both on crown roots (SDC) and L-type lateral roots (SDL) (Table 1, Additional file 1: Fig. S1). On the other hand, the density of L-types on crown roots (LDC) showed the opposite trend with low density in the nutrient solution. Similarly, the length of an individual L-type lateral on crown roots (LLC) was significantly reduced in nutrient solution where they reached only around 30 mm compared to an average of more than 110 mm in the rhizobox (Table 1). Despite the similarity of the other lateral root traits between rhizobox and field conditions, LLC was shorter in the field (56.9 mm), possibly indicating that excavating roots from the field using a spade damaged many L-type laterals. The length of S-type laterals was the least affected by screening environments but nutrient solution favored S-type elongation on crown roots but strongly reduced their elongation on L-type laterals.

**Table 1:** Genotypic difference (G) and experimental effect (E) on six lateral root traits evaluated from the nutrient solution, rhizobox, and low phosphorus field experiments in 2021

Root characteristic	Effect	Nutrient solution	G	RhizoBox	G	Low P field	G	G*E
L-type root density on crown root (LDC)	DJ123	2.8 (0.3)	a	3.2 (0.3)	b	2.9 (0.1)	ab	***
(roots per cm)	Nerica4	1.3 (0.3)	b	2.2 (0.3)	c	2.3 (0.7)	b	
	NDJ188	3.0 (0.0)	a	5.2 (0.3)	a	3.2 (0.3)	a	
	Average	2.4 (0.8)		3.5 (1.4)		2.8 (0.6)		
	E	B		A		AB		
S-type density on crown root (SDC)	DJ123	36.3 (0.4)	b	10.3 (0.3)	a	8.2 (1.7)	ab	***
(roots per cm)	Nerica4	12.9 (1.7)	c	4.7 (0.8)	b	6.9 (2.2)	b	
	NDJ188	43.4 (0.8)	a	11.0 (0.5)	a	10.1 (0.8)	a	
	Average	30.9 (15.9)		8.7 (3.1)		8.4 (2.0)		
	E	A		B		B		
S-type density on L-type (SDL)	DJ123	32.7 (1.2)	a	12.3 (0.3)	a	14.0 (3.7)	a	***
(roots per cm)	Nerica4	11.3 (0.4)	b	6.9 (0.4)	b	7.7 (0.8)	b	
	NDJ188	32.2 (0.3)	a	11.3 (0.3)	a	12.0 (1.0)	a	
	Average	25.4 (10.6)		10.1 (2.5)		11.2 (3.4)		
	E	A		B		B		
Single L-type length on crown root (LLC)	DJ123	28.5 (2.3)	ab	131.6 (23.3)	a	61.5 (5.5)	a	***
(mm)	Nerica4	24.5 (3.0)	b	60.7 (12.8)	b	53.9 (10.5)	a	
	NDJ188	37.2 (6.7)	a	140.4 (20.8)	a	59.3 (6.3)	a	
	Average	30.1 (6.8)		110.9 (41.4)		56.9 (5.3)		
	E	C		A		B		
Single S-type length on crown root (SLC)	DJ123	10.9 (1.7)	a	11.5 (0.9)	a	7.2 (0.2)	a	*
(mm)	Nerica4	7.5 (2.3)	a	2.7 (0.1)	b	5.5 (0.5)	b	
	NDJ188	8.8 (2.1)	a	8.5 (2.9)	a	6.3 (0.1)	ab	
	Average	9.0 (2.3)		7.6 (4.2)		6.3 (0.8)		
	E	A		AB		B		
Single S-type length on L-type (SLL)	DJ123	3.0 (0.8)	a	6.7 (0.4)	a	5.9 (2.4)	a	*
(mm)	Nerica4	2.9 (0.2)	a	2.0 (0.2)	b	3.5 (1.1)	a	
	NDJ188	2.9 (0.2)	a	4.4 (0.5)	ab	5.1 (1.5)	a	
	Average	2.9 (0.4)		4.4 (2.0)		4.8 (1.8)		
	E	B		A		A		

Mean phenotypic values are shown for each experimental condition and genotype. The number in parentheses indicate standard deviation. Two-way ANOVA was conducted, followed by Tukey–Kramer post-hoc test

Different alphabets indicate significant differences among groups at a = 0.05 significance threshold

For the interaction of environment and genotype, significance is indicated by * and ***, referring to the P < 0.05, and 0.001, respectively

Genotypic differences were detected for all root traits and Nerica4 had the lowest lateral root densities (Table 1, Additional file 1: Fig. S1). The difference between Nerica4 and backcross parent NDJ188 was the most pronounced in nutrient solution with around threefold differences for SDC and SDL, compared to slightly less than twofold differences in the field. Genotypic differences were generally much less pronounced for lateral root length and where they were significant, it was again Nerica4 with the lowest values. In nutrient solution, the only significant difference was for LLC where NDJ188 had about 50% longer L-types compared to Nerica4. In contrast, all length trait differences were highly significant in the rhizobox where LLC and SLL of NDJ188 were more than twice compared to Nerica4, which increased to a threefold difference for SLC (Table 1).

To clarify if root traits are affected by soil P availability, one additional experiment compared lateral root density and length in root samples taken from a P-fertilized (+P) compared to a P-deficient (− P) field at the Ishigaki experimental station. P deficiency had no significant effect on LDC, SDL and SLL but reduced SDC, LLC and SLC (Additional file 2: Table S1). There was no significant difference between genotypes for all six lateral root traits in +P field but genotypes differed significantly for LDC, SDL, and SLL at − P. For SDL Nerica4 showed a marked reduction under P deficiency whereas the other parents were not affected significantly. A reduction due to P deficiency was also seen for SLL and this was significant in Nerica4 and DJ123, while no change was seen in NDJ188.

### Variation and Heritability for Root Traits in the QTL Mapping Population

**Fig. 2:**
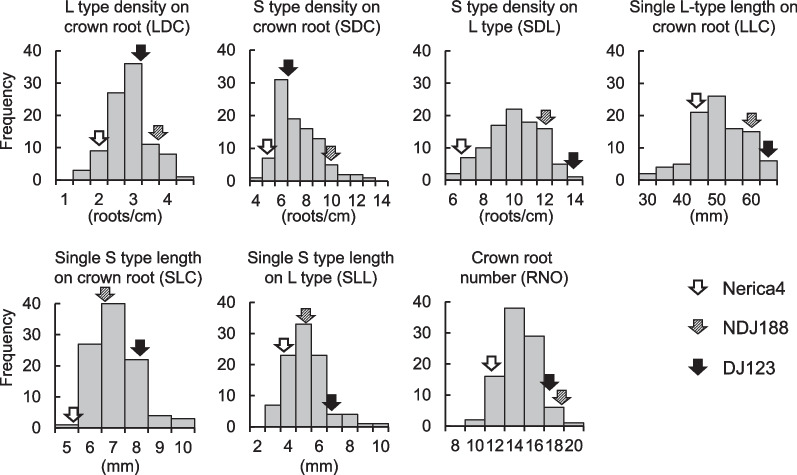
Frequency distribution of 98 BC1F5 lines for seven lateral root traits, including L-type density on the crown root (LDC), S-type density on the crown root (SDC), S-type density on L-type (SDL), Single S-type length on the crown root (SLC), single S-type length on L-type (SLL), and crown root number (RNO) from a low P field experiment in 2021 (Experiment 2)

The frequency distribution of 98 BC1F5 lines in field experiment 2 indicated transgressive segregation over parents Nerica4 and NDJ188 existed for all lateral root traits and for crown root number, which had been added as an additional trait as it contributes to root system size and P uptake (Fig. 2). Nerica4 was near the bottom end of the distribution for almost all root traits except for LLC where it was average. Lines of the mapping population showed the widest variation for SLL ranging almost fourfold from 2.5 to 9.4 mm, whereas other length traits showed lower variation with little over twofold ranges (27–65 mm for LLC, and 4.7–9.8 mm for SLC). All density traits varied roughly threefold, ranging from 1.5–4.2 roots cm^−1^ for LDC, 4.0–13 roots cm^−1^ for SDC, and 5.2–14 roots cm^−1^ for SDL (Fig. 2).

Broad sense heritability estimates based on progeny mean squares indicated S-type densities on crown and L-type laterals (SDC and SDL) to be highly heritable with H^2^ = 0.69 and H^2^ = 0.67, respectively (Table 2). Other root traits had moderate heritability with H^2^ of 0.43 (RNO), 0.46 (SLC), and 0.57 (SLL) while single L-type length on crown roots (LLC) had low heritability (H^2^ = 0.29).

**Table 2:** Estimates of variance components and broad sense heritability for seven root traits based on progeny mean squares of BC1F5 from experiment 2

Phenotype	Pr(> F)		EV	GV	PV	H^2^
L-type root density on crown root (LDC)	1.23E-08	***	0.87	0.97	1.84	0.52
S-type density on crown root (SDC)	1.17E-15	***	3.76	8.57	12.34	0.69
S-type density on L-type (SDL)	1.31E-14	***	4.6	9.5	14.2	0.67
Single L-type length on crown root (LLC)	1.80E-03	**	76.4	31.1	107.6	0.29
Single S-type length on crown root (SLC)	9.10E-07	***	0.68	0.59	1.28	0.46
Single S-type length on L-type (SLL)	2.14E-10	***	0.77	1.06	1.84	0.57
Root number (RNO)	4.69E-06	***	2.74	2.06	4.8	0.43

Environmental Variance (EV), Genotypic Variance (GV), Phenotypic Variance (PV), Broad Sense Heritability (H^2^)

Significant differences are indicated by ** and *** referring to P < 0.01, and 0.001, respectively

### QTL Detection and Phenotypic Effects

A linkage map containing 565 SNP markers was generated for the BC1F5 mapping population by aligning RAD-seq reads to the sequence of *tropical japonica* cultivar Azucena, and subsequent reduction of very closely linked markers. Since the mapping population was developed from a back-cross between Nerica4 and NDJ188 (from the Nerica4 × DJ123 population), the linkage map is characterized by large non-recombinant blocks on chromosomes 1, 2, 3, 10, 11 and 12 (Additional file 3: Fig. S3).

Composite interval mapping detected 5 QTL, one each for the traits LDC, SDC, SDL, SLL, and RNO (Table 3, Additional file 4: Fig. S4). No QTL was detected for SLC and LLC, which were traits for which less variation had been detected (Fig. 2). The most influential QTL was *qLDC5* on chromosome 5 with a LOD score of 11.4, explaining 46% of the variation for this trait. Variation of SDC and SLL was attributed to two distinct QTL on chromosome 1 (*qSDC1* and *qSLL1*) explaining 26% and 24% of the variation for the traits, respectively. They were 78 cM apart, which corresponded to a physical distance of almost 11.6 Mb. Smaller QTL were detected for SDL (*qSDL9*) and RNO (*qRNo9*) on chromosome 9 at positions 17 and 43 cM, respectively, which corresponds to a more than 4.3 Mb distance based on marker physical positions. For all QTL the alleles increasing lateral root density and length, or crown root number was from donor DJ123. Further examination of peaks in Additional file 4: Fig. S4 showed that each peak was specific for one trait without causing substantial effects on other traits, possibly indicating the absence of interactions among detected QTL.

**Table 3:** Putative QTL for lateral root traits detected in BC1F5 population (n = 98) grown in a field experiment on phosphorus-deficient soil in Tsukuba, 2021

Trait	QTL	Chromo some	Nearest marker^a^	Physical position (bp)	Position (cM)	Support interval	LOD	LOD threshold	*R*^*2*^	Additive effect	Allelic effect (%)	Positive allele
L-type density on crown root (LDC)	*qLDC5*	5	S05_27313585	26,276,202	60	51–75	11.4	3.6	0.46	0.43	33.8	DJ123
S-type density on crown root (SDC)	*qSDC1*	1	S01_29957378	29,269,714	180	176–188	6.3	4.0	0.17	0.79	22.9	DJ123
S-type density on L-type (SDL)	*qSDL9*	9	S09_8741627	8,182,160^b^	17	8–25	4.0	3.6	0.17	0.81	16.8	DJ123
Single S-type length on L-type (SLL)	*qSLL1*	1	S01_41579214	40,879,329	258	242–260	5.5	4.5	0.24	0.10	4.21	DJ123
Root Number (RNO)	*qRNO9*	9	S09_13090502	12,924,104	43	40–44	3.6	3.6	0.17	0.69	10.1	DJ123

*L*OD thresholds were determined through 1000-time permutations at α = 0.05

^a^Marker names indicate chromosomal location followed by their physical position based on the alignement with the Azucena genome (S09_8965532 indicates a SNP position at 8.965 Mb on chromosome 9). Physical position: correscponding position based on the Nipponbare referennce genome. Additive effects were estimated as the phenotypic effect of substituting one A (DJ123) allele with a B (Nerica4) allele with positive values indicating DJ123 alleles having a positive effect on phenotypic values. Allelic effects were calculated based on additive effects for the difference between homozygous individuals (AA vs. BB) and are given in % based on the overall mean of each trait

^b^A BLAST search identified a second hit in the Nipponbare genome at 7,069,632 bp on chromosome 9

### Effect of Root Traits on P Uptake

**Fig. 3:**
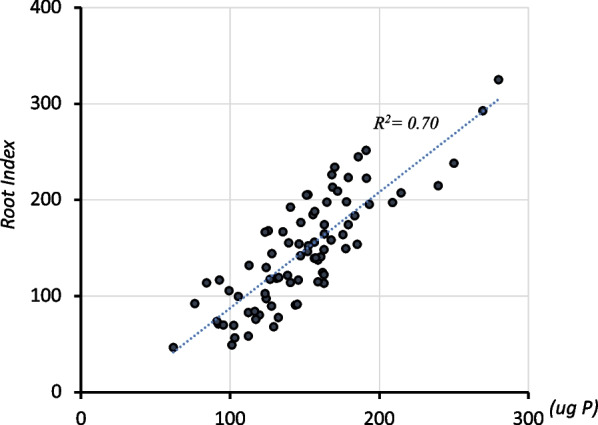
Association of shoot P content of the 98 lines of the QTL mapping population (experiment 2) with their Root Index calculated by multiplying the three most influential traits (RNO*LDC*SDL). Note: for better readability index values are given as 1/10 of their real value

Each of the seven traits analyzed here may contribute to root system size and subsequent P uptake, and to determine the most influential root traits, a stepwise linear regression analysis was conducted. The most influential traits were LDC and RNO, each explaining 38% of the variation for shoot P content among the 98 lines of the QTL mapping population (Table 4). A model using both traits increased the proportion of the variance explained to 58%. This increased further to 69% with the addition of SDL. Other traits were not included in the stepwise linear regression model or dropped from the model if they were chosen as a starting variable. Based on these results a simple Root Index was developed for each line by multiplying line values for RNO, LDC and SDL. The resultant index explained as much as 70% of the variation in shoot P content among the 98 lines of the QTL mapping population (Fig. 3).

**Table 4:** Contribution of root traits to shoot P content determined by stepwise linear regression analysis, based on data from the QTL mapping population (n = 98)

Trait	Individual *R*^*2*^	Cumulative *R*^*2*^	*P-*value
L-type density on crown root (LDC)	0.38	0.38	< 0.001
Root number (RNO)	0.38	0.58	< 0.001
S type density on L-type (SDL)	0.19	0.69	< 0.001
S type density on crown root (SDC)	–	0.69	0.48
Single L-type length on crown root (LLC)	–	0.69	0.47
Single S-type length on crown root (SLC)	–	0.69	0.79
Single S-type length on L-type (SLL)	–	0.69	0.22

### Confirmation of Allelic Effects at Main QTL in the BC1F6

The lines have contrasting root traits in the BC1F5 mapping experiment (experiment 2) were selected for further field experiments in 2022 (experiment 3) to confirm the effects of QTL for the most influential traits (LDC, RNO, SDL) identified through stepwise linear regression analysis. Dry shoot weight (DSW) was used as a proxy for shoot P content (the *R*^*2*^ between both traits was 0.83). Lines with positive DJ123 (AA) alleles at *qLDC5* (n = 10) and *qRNO9* (n = 19) had 15.8 and 17.3% higher DSW compared to lines with Nerica4 (BB) alleles, respectively (Table 5). For lines carrying AA alleles at both *qLDC5* and *qRNO9* (n = 5), the advantage in DSW over lines with BB alleles (n = 6) increased to 41.1%. We further attempted to evaluate the effect of *qSDL9* but its proximity to *qRNO9* on chromosome meant that very few recombinants between both QTL existed, and the lines combining *qLDC5* and *qRNO9* were identical to the lines combining all 3 positive alleles *(qLDC5* + *qRNO9* + *qSDL9*). The 41.1% advantage in DSW seen for *qLDC5* + *qRNO9* would therefore include positive effects of *qSDL9* (Table 5).

**Table 5:** Mean dry shoot weight (DSW) of contrasting groups of lines selected for being homozygous for the DJ123 (AA) or Nerica4 (BB) allele at QTL *qLDC5, qSDL9,* and *qRNO9*. Data from experiment 3 in 2022

QTL name	Allele	DSW (mg)	SD	n
*qLDC5*	AA	390.8^ab^	79.2	10
	BB	337.7^bc^	62	18
	%	**15.8**		
*qRNO9*	AA	370.2^ab^	20.4	19
	BB	315.8^bc^	31.6	10
	%	**17.3**		
*qLDC5* + *qRNO9*	AA	433.4^a^	42.2	5
	BB	307.5^bc^	17	6
	%	**41.1**		
*qLDC5* + *qSDL9* + *qRNO9*	AA	433.4^a^	42.2	5
	BB	307.5^bc^	17	6
	%	**41.1**		
Nerica4	BB	287.9^c^	51.1	10
DJ123	AA	414.3^a^	42.4	19
	%	**43.9**		

Significance difference of mean are indicated by different letter refer to the P < 0.05 by Tukey HSD All-Pairwise Comparisons Test

**Fig. 4:**
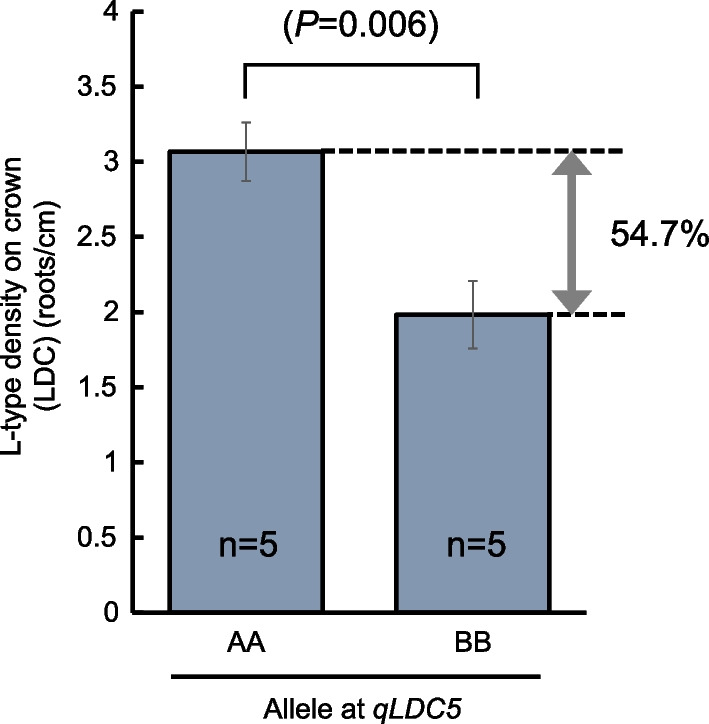
Confirmation of allelic effects at *qLDC5* in the BC1F6 generation by measuring L-type lateral root density on root samples excavated from the low-P field in Tsukuba in 2022. Different letters indicate significant differences and “n” indicates the number of lines classified into each allelic group

QTL effects were further confirmed by measuring L-type lateral root density on root samples excavated from the low-P field in Tsukuba in 2022. Five representative lines of the positive and negative allele at *qLDC5* were evaluated. Lines with positive AA alleles at *qLDC5* had on average 3.07 (± 0.19) L-type roots cm^−1^ crown length versus 1.98 (± 0.22) L-type roots cm^−1^ in lines with the BB allele, which corresponds to an increase of 54.7% (Fig. 4).

Using the model of Gonzalez et al. (2021), we simulated the hypothetical replacement of positive by negative alleles at *qLDC5*, *qRNO9* and *qSDL9* and estimated changes in P uptake caused by estimated QTL effects. Lines with Nerica4 alleles at *qRNO9* were estimated to have 10.1% fewer crown roots and this had a slightly stronger effect on reducing total root system length (− 12.1%; Table 6). As a result, simulated P uptake at day 30 was 11.8% lower in lines with BB alleles at *qRNO9* (Table 6). The allelic effect of *qLDC5* (− 33.8%) was stronger than that of *qRNO9* and this reduced P uptake by 20.6%, whereas the effect of *qSDL9* on P uptake was minimal with a 3.9% reduction. For the combination of all three allelic effects the model simulated a reduction in P uptake of 26.4% (Table 6).

**Table 6:** Simulated changes in root development and P uptake caused by root trait differences conferred by the main QTL for RNO, LDC and SDL

Trait	Base	RNO (− 10.1%)*		LDC (− 33.8%)*		SDL (− 16.8%)*		RNO + LDC + SDL	
		Value	%	Value	%	Value	%	Value	%
Total root surface area (RSA)	257.5	230.1	89.3	222.0	86.2	251.4	97.6	206.6	80.2
Total root length (RL)	2888.8	2537.8	87.9	2079.2	72.0	2717.5	94.1	1916.3	66.3
RL crown roots	648.3	585.5	90.3	638.5	98.5	638.0	98.4	590.2	91.0
RL L-type lateral roots	1012.4	887.1	87.6	608.2	60.1	987.4	97.5	582.7	57.6
RL S-type lateral roots	1228.1	1065.2	86.7	832.6	67.8	1092.1	88.9	743.4	60.5
P uptake day 30 (μg P)	31.2	27.5	88.2	24.8	79.6	30.0	96.1	23.0	73.6

The change from having positive DJ123 alleles to negative Nerica4 alleles were simulated, hence a reduction in phenotypic values causes a reduction in root growth and P uptake

*10.1%, 33.8% and 16.8% are the allelic effects calculated based on the additive effect of the mean determined in the QTL mapping for RNO, LDC and SDL, respectively (see Table 3)

## Discussion

### Effect of Screening Conditions on Lateral Root Growth

Lateral root elongation and branching patterns may be influenced by the growth medium in which they develop. Such effects were detected for the density and length of root hairs: in nutrient solution root hair development was concentrated on crown roots but was almost completely suppressed on lateral roots, whereas soil-grown lateral roots were densely covered by root hairs (Nestler et al. 2016). To determine whether similar effects can be observed for lateral root development, their length and density were compared between three genotypes grown in soil or nutrient solution. Generally, the ability to detect genotypic differences was not affected for lateral root density traits, despite 2–3 fold higher S-type densities in nutrient solution compared to soil-grown roots. This contrasted with S-type lateral root length for which genotypic differences could no longer be detected in nutrient solution whereas they were more evident in soil-grown roots, especially in the rhizobox. We conclude that if the objective is to detect genotypic variation for lateral root density alone, a nutrient solution screen would be sufficient. However, if lateral root length traits are of interest, screening needs to be done with soil-grown plants. Here the rhizobox appears the preferable and more precise method, especially for L-type length, which was strongly reduced in field-grown plants likely as a result of damage during the shallow root excavation with a spade. The problem of L-type lateral damage may be particularly due to the tendency of long L-types to develop at depth below 10–15 cm (Additional file 5: Fig. S2; De Bauw et al. 2020), which were not recovered fully in the shallower sampling done here. Despite the obvious advantages of the rhizobox, the large number of lines required in QTL mapping studies typically necessitates a simpler and more economical screen in the field or in some large soil-filled containers.

The nutrient solution suppressed the elongation of both L-type and L-type-originating S-type lateral roots but not that of S-type laterals growing on crown roots. This is similar to the suppression of root hair development on lateral roots in nutrient solution seen by Nestler et al. (2016) and may indicate a general preference for crown roots over L-type laterals in nutrient solution that is not mirrored in plants grown in aerobic soil.

### QTL Detection and Confirmation

This study identified four QTL for lateral root traits on chromosomes 1, 5 and 9 with *qLDC5* having a major effect. A subsequent experiment detected a 54.7% difference in L-type density between lines contrasting at this locus, thus confirming its large phenotypic effect. The confidence interval for *qLDC5* is between 24.9 and 29.2 Mb on chromosome 5 (peak at 27.3 Mb) and several root-related QTL had been identified in this chromosomal region. Price et al., (2002) detected a QTL for deep root weight and maximum root length in the Bala x Azucena mapping population. Similarly, QTL for maximum root length (*qMRL5-1*), root dry weight (*qRDM5-1*), and shoot or total biomass were detected within the confidence interval of *qLDC5* (Cui et al 2002; Lian et al. 2005). These studies did not determine lateral root traits, and it is therefore not possible to discern whether the reported loci mainly affect crown root traits or had additional effects on lateral root development. Where L-type lateral root traits have been investigated in detail, L-type lateral root density QTL were detected on chromosome 8, and for total lateral root density on chromosome 12 (Niones et al. 2015). Furthermore, Wang et al. (2018) identified a QTL on chromosome 11 (*qTIPS-11*) associated with a 32.4% increase in lateral root number without distinguishing S-type and L-type lateral roots.

The three QTL identified for S-type density or length on chromosomes 1 (*qSDC1* and *qSLL1*) and 9 (*qSDL9*) had smaller phenotypic effects compared to *qLDC5*. Previous studies reported QTL for root traits related to crown root development, such as maximum root length and thickness, root penetration or deep root weight in similar region as *qSDL9* (Kamoshita et al. 2002; Lian et al. 2005), *qSDC1* (Yadav et al. 1997) and *qSLL1* (Ali et al. 2000; Lian et al. 2005). One QTL for a root branching index, which estimated the proportion of lateral root length over total root length, was detected at 23.3 Mb on chromosome 1 (Horii et al. 2006) at a considerable distance from *qSDC1* (29.9 Mb).

The locus increasing crown root number on chromosome 9 (*qRNO9*) had already been identified as *qPef-9* in the same Nerica4 × NDJ188 population used here, but in the BC1F3 generation (Ranaivo et al. 2022). Its interval between 12.71 and 13.69 Mb provides a perfect overlap with the interval identified here. That study had identified a second locus (*qPef9-2*) in close proximity (9.64–10.76 Mb on chromosome 9), and confirmatory studies with contracting introgression lines showed significant positive and very similar effects for both *qPef-9* and *qPef9-2*, not allowing for clearly establishing whether both QTL were two distinct loci or one single poorly defined locus. Here a second QTL *qSDL9* (7.65–10.07 Mb on chromosome 9) was identified in proximity to *qRNO9* and as in the study by Ranaivo et al. (2022), an insufficient number of recombinants between both loci did not allow us to establish whether *qSDL9* and *qRNO9* were indeed two distinct loci. Further fine-mapping will be needed to resolve this question. Nevertheless, we could confirm that the joint presence of *qSDL9* and *qRNO9* improved growth under P deficiency and that combining these with *qLDC5* had an even larger positive effect that should be explored further in rice improvement.

### Potential Auxin-Related Candidate Genes Underlying Detected QTL

Previous studies identified genes associated with lateral root formation or development in rice. Auxin is one of the major factors affecting the root morphology (Meng et al. 2019). Aux/IAA family proteins interact with auxin response factor (ARF) proteins and mediate auxin-induced transcriptional changes. Previous investigations suggested that some members of Aux/IAA family proteins are involved in the regulation of lateral root growth in rice; IAA31 is the first Aux/IAA family member shown to be involved in lateral root formation, through an induced expression approach (Nakamura et al. 2006). Mutants for *IAA11*, *IAA13* and *IAA23* significantly reduced lateral root number on crown roots (Ni et al. 2011; Kitomi et al. 2012; Zhu et al. 2012). Overexpression of *IAA1* reduced the number of crown roots but increased lateral root density (Song et al. 2009). Intriguingly, the Aux/IAA family gene *IAA19*, which is phylogenetically close to *IAA1*, *IAA11*, *IAA13* and *IAA23* (Additional file 6: Fig. S5), is located at 27.84 Mb on chromosome 5 and therefore in proximity to *qLDC5*. Further, the closest *IAA19* homologue, *IAA5*, is located at 27.78 Mb on chromosome 1, while the more distantly related *IAA6* is found at 30.98 Mb on chromosome 1. Both positions flank the peak location of *qSDC1*. A potential role of these IAA homologues co-localizing with *qLDC5* and *qSDC1* should be investigated further.

Not an IAA homologue but induced by auxin in roots, the cytokinin oxidase/dehydrogenase CKX4 co-localizes with qSLL1 and was found to be abundantly expressed in lateral roots (Gao et al. 2014). Furthermore, a dominant mutant for CKX4 exhibited higher sensitivity to exogenous auxin and showed increased crown root number and length (Gao et al. 2014), making it another possible candidate for further consideration. No previously identified candidate gene affecting root morphology was detected close to the peak position of* qSDL9* and *qRNO9*.

### Role of Detected QTL for P Uptake

Genotypic differences in the ability to take up P from the soil are largely due to differences in root size with smaller additional effects due to higher P uptake efficiency per unit root size in some genotypes (Mori et al. 2016; Wissuwa et al. 2020). The size of the root system is determined by crown root length and the seven traits measured in this study: the number of crown roots (RNO) determines the number of main root axes from which all other roots branch off; L-type density on crown roots (LDC) and single L-type length on crown roots (LLC) then provide the next order of branching; while the density and length of S-type roots on crown roots (SDC and SLC) or on L-type laterals (SDL and SLL) determines the total size of fine root structures with a large contribution to total root system length but a much smaller contribution to total root surface area due to the small diameter of S-type lateral roots (Gonzalez et al. 2021). Here we detected QTL for five of these seven traits with *qLDC5* being considered a major QTL.

It is of practical relevance to consider the possible contribution of each trait and QTL to total P uptake. The stepwise linear regression identified LDC and RNO as two of the most influential traits with an additional small positive effect of SDL, and the simple Root Index derived from the multiplication of phenotypic values of all lines of the QTL mapping population for these three traits, explained 70% of the variation in measured P uptake in the mapping population. Crown root length was not measured in this study but if included as a fourth trait, would have likely further increased the predictive ability of the Root Index. The importance of having more of the larger crown and L-type roots is expected for two reasons: their larger surface area will directly enhance P uptake (Gonzalez et at. 2021), but both types are also parent roots of S-type laterals, thus more parent root axes will have carry-over effects on P uptake through a larger total number of S-type roots developing on the parent root (Kuppe et al. 2022). It is less clear why S-type density on L-type (SDL) was more influential than S-type density on crown roots (SDC). Possible reasons are the slightly higher genotypic variation for SDL among lines of the mapping population, and the higher proportion of total root system length contributed by L-type roots compared to crown roots (Gonzalez et al. 2021).

Given that most of our understanding of the role of different root types is derived from modeling studies, we simulated the effect of replacing positive with negative alleles at *qLDC5*, *qRNO9* and *qSDL9* on P uptake using the model of Gonzalez et al. (2021). Allelic effects were much larger for LDC (33.8%) than for RNO (10.1%) but their simulated effect on P uptake was much closer (20.4% vs. 11.8%, respectively), which was caused by the larger knock-on effects of reducing crown roots, which will reduce L-type and S-type lateral root numbers, compared to the reduction in L-type root density that will only affect the S-type laterals developing on these L-type lateral roots. Completely without knock-on effects is the reduction in SDL, which explains the small effect on P uptake of this trait simulated by the model of Gonzalez et al. (2021).

This model does not take P solubilization in the rhizosphere into account and therefore underestimates P uptake by fine root structures such as S-type laterals and root hairs, which may access P solubilized by larger roots at a distance exceeding the effective range for diffusion (Kuppe et al. 2022). It is thus expected that the contribution of SDL to P uptake of field-grown plants would increase, and that would increase the joint effect of combining all three QTL from the current estimate of 26.4% (P uptake) to a value closer to the observed biomass effect of 41.1% (Table 5). An additional reason for expecting larger long-term effects compared to our conservative model estimates is a different kind of knock-on effect: when P deficiency limits plant growth including root development, a portion of the additional P uptake provided by an efficiency mechanism (here: the joint effect of combining all three QTL) will be re-invested in better root development, which will accelerate subsequent P uptake (Wissuwa 2003).

## Conclusions

This study identified four novel QTL for lateral root density and length together with a crown root number QTL and *qLDC5* for L-type lateral root density had a major effect, both in explaining a very high proportion of the phenotypic variation for the trait as well as in terms of improving P uptake. This QTL should be targeted in upland rice breeding after further fine-mapping has provided more tightly linked markers. Pyramiding *qLDC5* with the crown root number locus *qRNO9* and *qSDL9* for S-type lateral density on L-type laterals seems feasible as trade-offs between traits are not expected, given that the Root Index combining these three traits was highly predictive of P uptake. Furthermore, both *qRNO9* and *qSDL9* are located in close proximity on chromosome 9 and could be introgressed simultaneously during marker-assisted selection. While phenotypic selection during variety development would be feasible for crown root number, the costs of doing so reliably would be prohibitive for lateral root density traits and markers identified here therefore provide the first opportunity to incorporate such traits into a breeding program.

### Supplementary Information

**Additional file 1**. **Fig. S1**: Differences in lateral root densities of three parental genotypes (DJ123, Nerica4, and NDJ188) grown in nutrient solution (**a**) or in low-P soil (**b**). In nutrient solution, S-type lateral roots emerge at high density but only on crown roots with much less S-type development on L-type laterals compared to soil-grown roots.

**Additional file 2**. **Table S1**. Genotypic differences in lateral root traits under normal input (+P) and low input (−P) field conditions grown in Ishigaki in 2021.

**Additional file 3**. **Fig. S3**. A linkage map containing 565 SNP markers was generated for the BC1F5 mapping population. Markers were initially ordered based on their physical map positions, but the final order was determined after applying the ripple function in Rqtl. Genetic distances were estimated in cM using the Kosambi option.

**Additional file 4**. **Fig. S4**. Positions of five detected QTL including qSDC1 (dark blue dashed line), and qSLL1 (orange dashed line), qLDC5 (green dashed line), qSDL9 (grey dashed line), qRNO9 (pink dashed line) on chromosomes 1, 5, and 9 as indicated by the QTL analysis using QGENE. Results indicate that each peak was specific for one trait without overlapping near-significant effects for other traits, possibly implying the absence of interactions between QTL detected.

**Additional file 5**. **Fig. S2**. Differences in single L-type length on crown roots (LLC) between three parental genotypes (DJ123, Nerica4, NDJ188) from the rhizobox experiment. **a** Scanned photos of a single crown root. The root was divided into 2-3 segments to fit within the scanned area. Segments on the right are proximal to the crown and on the left are proximal to the tip region. L-type lateral roots developed more densely at medium depth. **b** A significant 2-fold difference in LLC was detected between DJ123, NDJ188, and Nerica4 (P<0.01).

**Additional file 6**. **Fig.** **S5**. Phylogenetic tree of the AUX/IAA family genes inferred using the Neighbor-Joining method in MEGA X software (Stecher et al. 2020). Genes previously suggested to be involved in lateral root traits are shown by magenta circles, while genes that were found in close proximity with QTL from the current study are shown by blue circles.

## Data Availability

The sequencing dataset generated during the current study are available in the NCBI SRA repository (accession number PRJNA870975; https://www.ncbi.nlm.nih.gov/bioproject/933277). The other data supporting the findings of this study are available within the paper and within its supplementary materials published online.

## Abbreviations

LDC: L-type density on crown root
RNO: Crown root number
SDC: S-type density on crown root
SDL: S-type density on L-type
SLL: Single S-type length on L-type

## References

[CR100] Ali ML, Pathan MS, Zhang J, Bai G, Sarkarung S, Nguyen HT (2000). Mapping QTLs for root traits in a recombinant inbred population from two indica ecotypes in rice. Theor Appl Genet.

[CR1] Baird NA, Etter PD, Atwood TS, Currey MC, Shiver AL, Lewis ZA, Selker EU, Cresko WA, Johnson EA (2008). Rapid SNP discovery and genetic mapping using sequenced RAD markers. PLoS ONE.

[CR2] Bolger AM, Lohse M, Usadel B (2014). Trimmomatic: a flexible trimmer for Illumina sequence data. Bioinformatics.

[CR3] Bradbury PJ, Zhang Z, Kroon DE, Casstevens TM, Ramdoss Y, Buckler ES (2007). TASSEL: software for association mapping of complex traits in diverse samples. Bioinformatics.

[CR4] Broman KW, Wu H, Sen S, Churchill GA (2003). R/qtl: QTL mapping in experimental crosses. Bioinformatics.

[CR5] Cui H, Peng B, Xing Z, Xu G, Yu B, Zhang Q (2002). Molecular dissection of seedling-vigor and associated physiological traits in rice. Theor Appl Genet.

[CR6] Danecek P, Auton A, Abecasis G, Albers CA, Banks E, DePristo MA, Handsaker RE, Lunter G, Marth GT, Sherry ST, McVean G, Durbin R (2011). The variant call format and VCFtools. Bioinformatics.

[CR7] De Bauw P, Mai TH, Schnepf A, Merckx R, Smolders E, Vanderborght J (2020). A functional-structural model of upland rice root systems reveals the importance of laterals and growing root tips for phosphate uptake from wet and dry soils. Ann Bot.

[CR8] FAO (United Nations Food and Agriculture Organization) “Rice is Life” (PDF) (2004) Archived (PDF) from the original on November 10, 2011. Accessed on 21 Nov 2011

[CR9] Gao S, Fang J, Xu F (2014). Cytokinin oxidase/dehydrogenase4 integrates cytokinin and auxin signaling to control rice crown root formation. Plant Physiol.

[CR10] Gonzalez D, Postma J, Wissuwa M (2021). Cost-benefit analysis of the upland-rice root architecture in relation to phosphate: 3D simulations highlight the importance of S-type lateral roots for reducing the pay-off time. Front Plant Sci.

[CR11] Hemamalini GS, Shashidhar HE, Hittalmani S (2000). Molecular marker assisted tagging of morphological and physiological traits under two contrasting moisture regimes at peak vegetative stage in rice (*Oryza sativa* L.). Euphytica.

[CR12] Horii H, Nemoto K, Miyamoto N, Harada J (2006). Quantitative trait loci for adventitious and lateral roots in rice. Plant Breed.

[CR13] Joehanes R, Nelson JC (2008). QGene 4.0, an extensible Java QTL-analysis platform. Bioinformatics.

[CR14] Kamoshita A, Wade J, Ali L, Pathan S, Zhang J, Sarkarung S, Nguyen T (2002). Mapping QTLs for root morphology of a rice population adapted to rainfed lowland conditions. Theor Appl Genet.

[CR15] Kitomi Y, Inahashi H, Takehisa H (2012). OsIAA13-mediated auxin signaling is involved in lateral root initiation in rice. Plant Sci.

[CR16] Kobayashi M, Ohyanagi H, Takanashi H, Asano S, Kudo T, Kajiya KH (2017). Heap: A highly sensitive and accurate SNP detection tool for low-coverage high-throughput sequencing data. DNA Res.

[CR17] Kuppe CW, Kirk GJD, Wissuwa M, Postma JA (2022). Rice increases phosphorus uptake in strongly sorbing soils by intra-root facilitation. Plant Cell Environ.

[CR18] Li H, Durbin R (2009). Fast and accurate short read alignment with Burrows-Wheeler transform. Bioinformatics.

[CR19] Li H, Handsaker B, Wysoker A, Fennell T, Ruan J, Homer N, Marth G, Abecasis G, Durbin R (2009). The sequence alignment/map format and SAMtools. Bioinformatics.

[CR20] Lian X, Xing Y, Yan H, Xu C, Li X, Zhang Q (2005). QTLs for low nitrogen tolerance at seedling stage identified using a recombinant inbred line population derived from an elite rice hybrid. Theor Appl Genet.

[CR21] Meng F, Xiang D, Zhu J (2019). Molecular mechanisms of root development in rice. Rice.

[CR22] Money D, Gardner K, Migicovsky Z, Schwaninger H, Zhong GY, Myles S (2015). LinkImpute: fast and accurate genotype imputation for nonmodel organisms. G3 Genes Genomes Genetics.

[CR23] Mori A, Fukuda T, Vejchasarn P, Nestler J, Pariasca-Tanaka J, Wissuwa M (2016). The role of root size versus root efficiency in phosphorus acquisition in rice. J Exp Bot.

[CR24] Nakamura A, Umemura I, Gomi K (2006). Production and characterization of auxin-insensitive rice by overexpression of a mutagenized rice IAA protein. Plant J.

[CR25] Nestler J, Keyes SD, Wissuwa M (2016). Root hair formation in rice (*Oryza sativa* L.) differs between root types and is altered in artificial growth conditions. J Exp Bot.

[CR26] Ni J, Wang G, Zhu Z (2011). OsIAA23-mediated auxin signaling defines postembryonic maintenance of QC in rice. Plant J.

[CR27] Niones JM, Inukai Y, Suralta RR, Yamauchi A (2015). QTL associated with lateral root plasticity in response to soil moisture fluctuation stress in rice. Plant Soil.

[CR28] Ouellette LA, Reid RW, Blanchard SG, Brouwer CR (2018). LinkageMapView-rendering high-resolution linkage and QTL maps. Bioinformatics.

[CR29] Popat R, Patel R, Parmar D (2020) Variability: genetic variability analysis for plant breeding research. R package version 0.1.1. http://CRAN.R-project.org/package=variability.

[CR30] Price AH, Steele KA, Moore BJ, Jones RGW (2002). Upland rice grown in soil-filled chambers and exposed to contrasting water-deficit regimes: II. Mapping quantitative trait loci for root morphology and distribution. Field Crops Res.

[CR31] Prodhan MA, Pariasca-Tanaka J, Ueda Y (2022). Comparative transcriptome analysis reveals a rapid response to phosphorus deficiency in a phosphorus-efficient rice genotype. Sci Rep.

[CR32] Ranaivo HN, Lam DT, Ueda Y, Tanaka JP, Takanashi H, Ramanankaja L, Razafimbelo T, Wissuwa M (2022). QTL mapping for early root and shoot vigor of upland rice (*Oryza sativa* L.) under P deficient field conditions in Japan and Madagascar. Front Plant Sci.

[CR33] Rebouillat A, Dievart A, Verdeil JL, Escoute J, Giese G, Breitler JC, Gantet P, Espeout S, Guiderdoni E, Périn C (2009). Molecular genetic of rice root development. Rice.

[CR34] Saito K, Vandamme E, Johnson JM (2019). Yield-limiting macronutrients for rice in sub-Saharan Africa. Geoderma.

[CR35] Schneider CA, Rasband WS, Eliceiri KW (2012). NIH Image to ImageJ: 25 years of image analysis. Nat Methods.

[CR36] Song Y, You J, Xiong L (2009). Characterization of OsIAA1 gene, a member of rice Aux/IAA family involved in auxin and brassinosteroid hormone responses and plant morphogenesis. Plant Mol Biol.

[CR37] Stecher G, Tamura K, Kumar S (2020). Molecular evolutionary genetics analysis (MEGA) for macOS. Mol Biol Evol.

[CR101] Vandamme E, Wissuwa M, Rose TJ, Dieng I, Dramé KN, Fofana M, Senthilkumar K, Venuprasad R, Jellow D, Segda Z, Suriyagoda L, Sirisena D, Kato Y, Saito K (2016). Genotypic variation in grain P loading across diverse rice growing environments and implications for field P balances. Front Plant Sci.

[CR38] Wang F, Longkumer T, Catausan SC (2018). Genome-wide association and gene validation studies for early root vigour to improve direct seeding of rice. Plant Cell Environ.

[CR39] Wissuwa M (2003). How do plants achieve tolerance to phosphorus deficiency? Small causes with big effects. Plant Physiol.

[CR40] Wissuwa M, Kondo K, Fukuda T, Mori A, Rose MT, Pariasca-Tanaka J, Kretzschmar T, Haefele SM, Rose TJ (2015). Unmasking novel loci for internal phosphorus utilization efficiency in rice germplasm through Genome-Wide Association Analysis. PLoS ONE.

[CR41] Wissuwa M, Gonzalez D, Watts-Willliams SJ (2020). The contribution of plant traits and soil microbes to phosphorus uptake from low-phosphorus soil in upland rice varieties. Plant Soil.

[CR42] Yadav R, Courtois B, Huang N, McLaren G (1997). Mapping genes controlling root morphology and root distribution in a doubled-haploid population of rice. Theor Appl Genet.

[CR43] Yoshida S, Forno DA, Cock JH, Gomez KA (1972) Laboratory manual for physiological studies of rice (2nd edn). International Rice Research Institute, pp 1–70.

[CR44] Zhu ZX, Liu Y, Liu SJ (2012). A gain-of-function mutation in OsIAA11 affects lateral root development in rice. Mol Plant.

