# Supplementary figures and images for: Novel QTL for Lateral Root Density and Length Improve Phosphorus Uptake in Rice (Oryza sativa L.)
Source: Rice (N Y). 2023 Aug 24;16:37. doi: 10.1186/s12284-023-00654-z (PMC10449758; doi:10.1186/s12284-023-00654-z)

DJ123

Nerica4

NDJ188

a

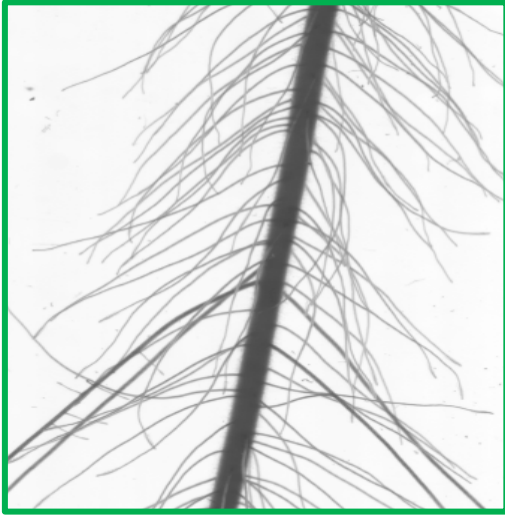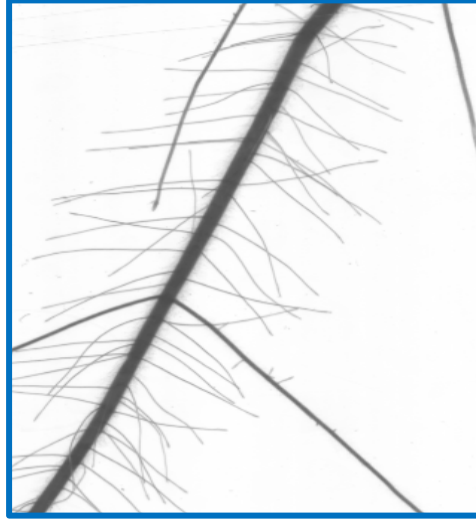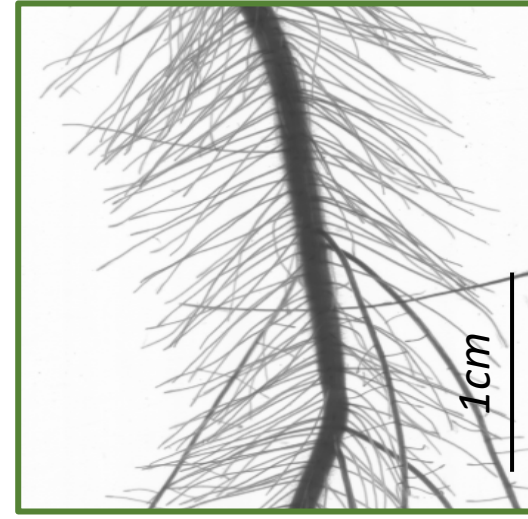

b

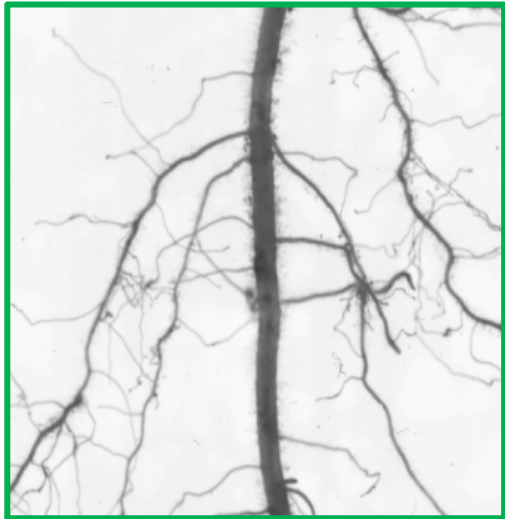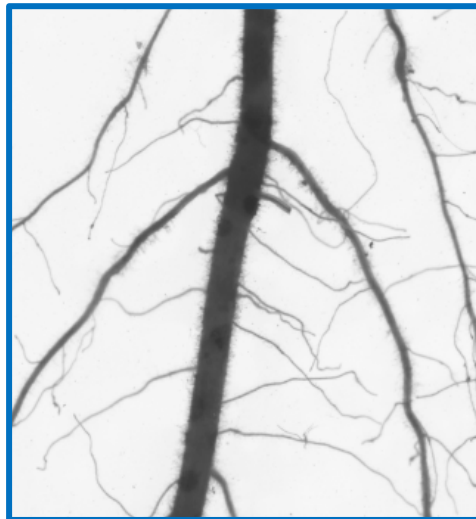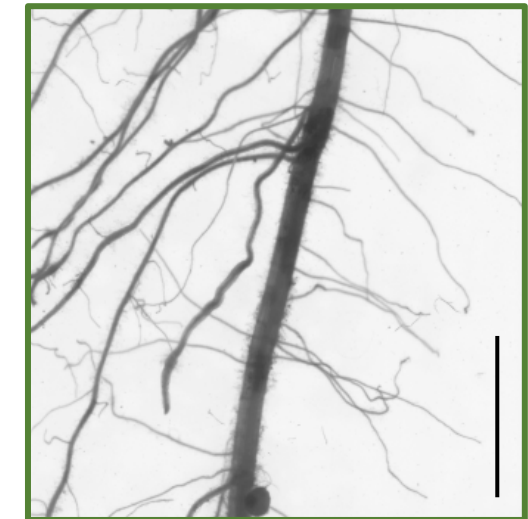

Supplement: Supplementary file 1 — Additional file 1. Fig. S1: Differences in lateral root densities of three parental genotypes (DJ123, Nerica4, and NDJ188) grown in nutrient solution (a) or in low-P soil (b). In nutrient solution, S-type lateral roots emerge at high density but only on crown roots with much less S-type development on L-type laterals compared to soil-grown roots. [file 12284_2023_654_MOESM1_ESM.pdf]

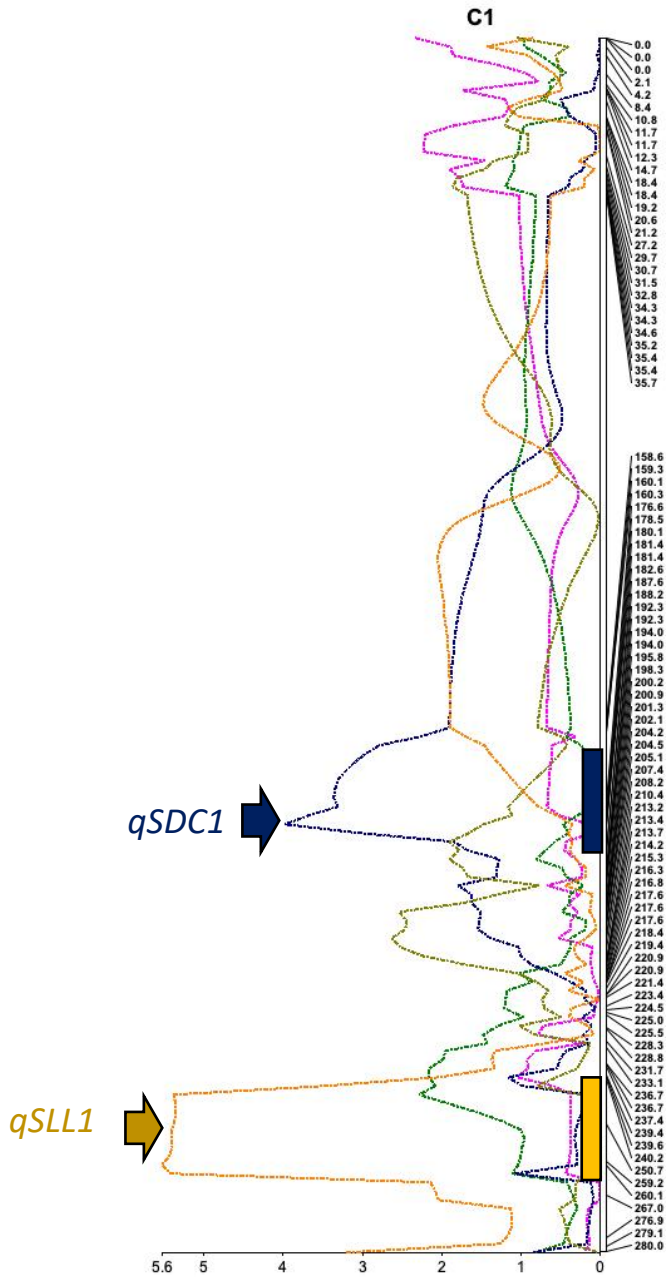

Rno  
 LDC  
 SDC  
 SDL  
 SLL

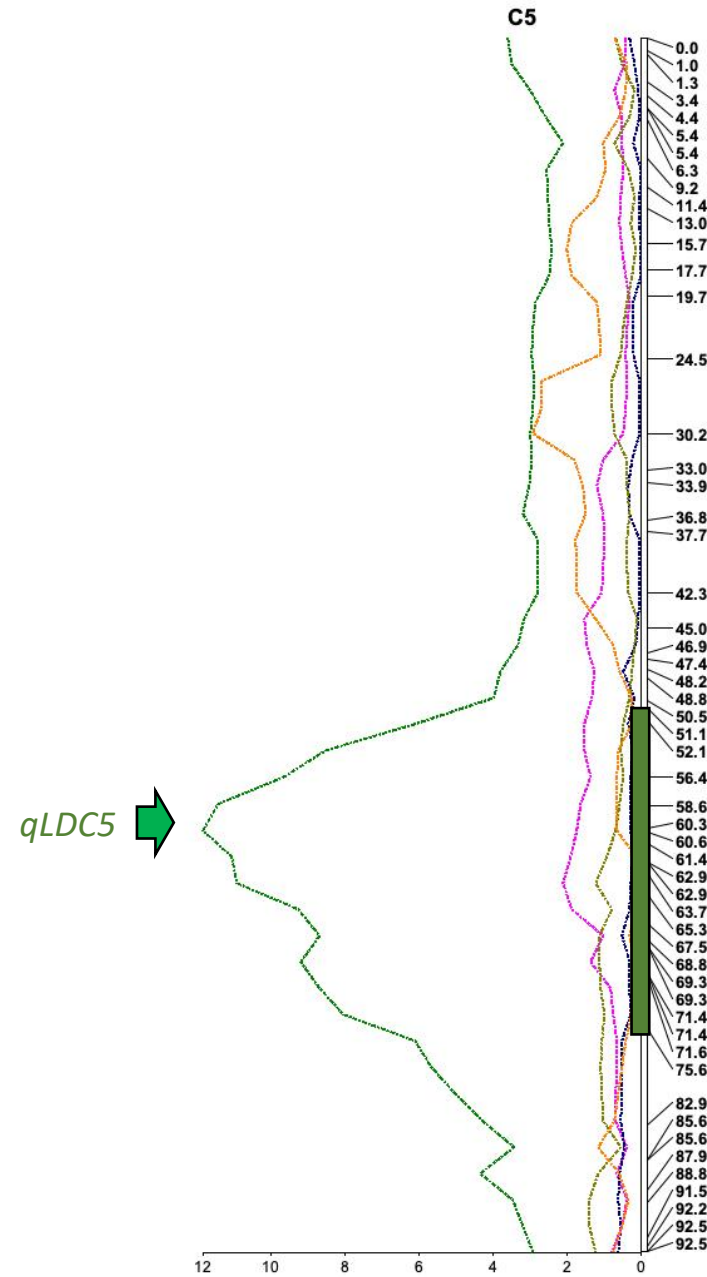

Rno  
 LDC  
 SDC  
 SDL  
 SLL

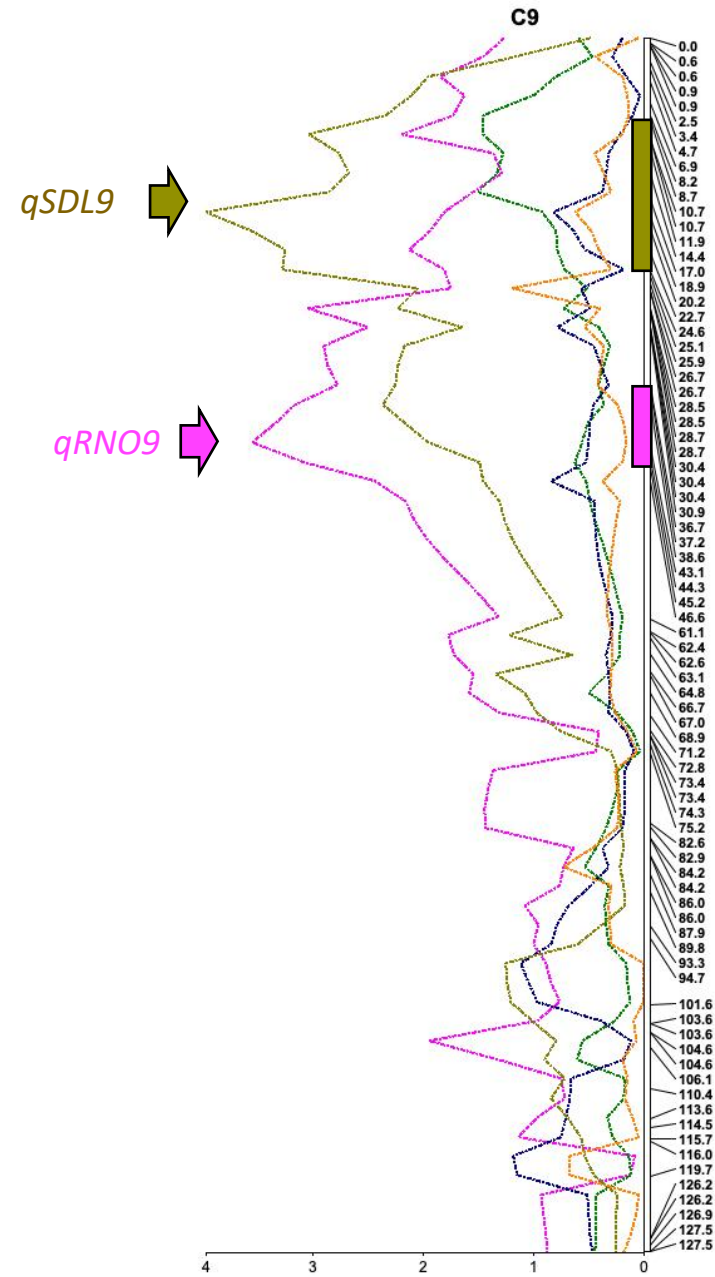

Rno  
 LDC  
 SDC  
 SDL  
 SLL

Supplement: Supplementary file 4 — Additional file 4. Fig. S4. Positions of five detected QTL including qSDC1 (dark blue dashed line), and qSLL1 (orange dashed line), qLDC5 (green dashed line), qSDL9 (grey dashed line), qRNO9 (pink dashed line) on chromosomes 1, 5, and 9 as indicated by the QTL analysis using QGENE. Results indicate that each peak was specific for one trait without overlapping near-significant effects for other traits, possibly implying the absence of interactions between QTL detected. [file 12284_2023_654_MOESM4_ESM.pdf]

**a.**

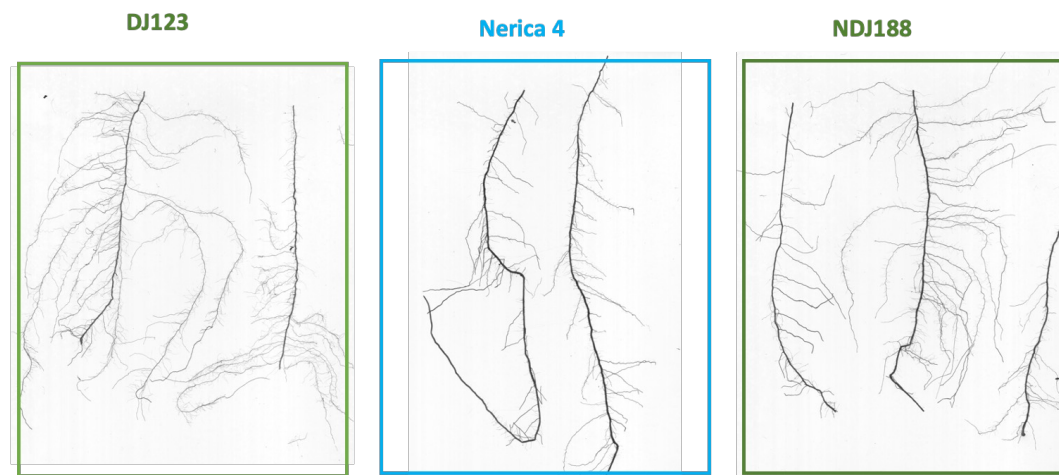

**b.**

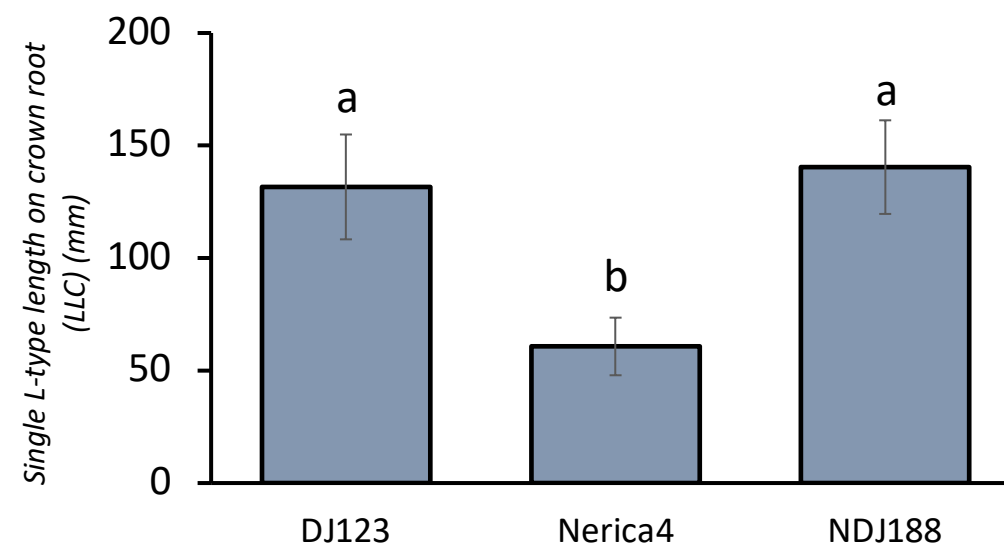

Supplement: Supplementary file 5 — Additional file 5. Fig. S2. Differences in single L-type length on crown roots (LLC) between three parental genotypes (DJ123, Nerica4, NDJ188) from the rhizobox experiment. a Scanned photos of a single crown root. The root was divided into 2-3 segments to fit within the scanned area. Segments on the right are proximal to the crown and on the left are proximal to the tip region. L-type lateral roots developed more densely at medium depth. b A significant 2-fold difference in LLC was detected between DJ123, NDJ188, and Nerica4 (P<0.01). [file 12284_2023_654_MOESM5_ESM.pdf]

- Closely located to QTL in the current study
- Previously known to affect LR formation

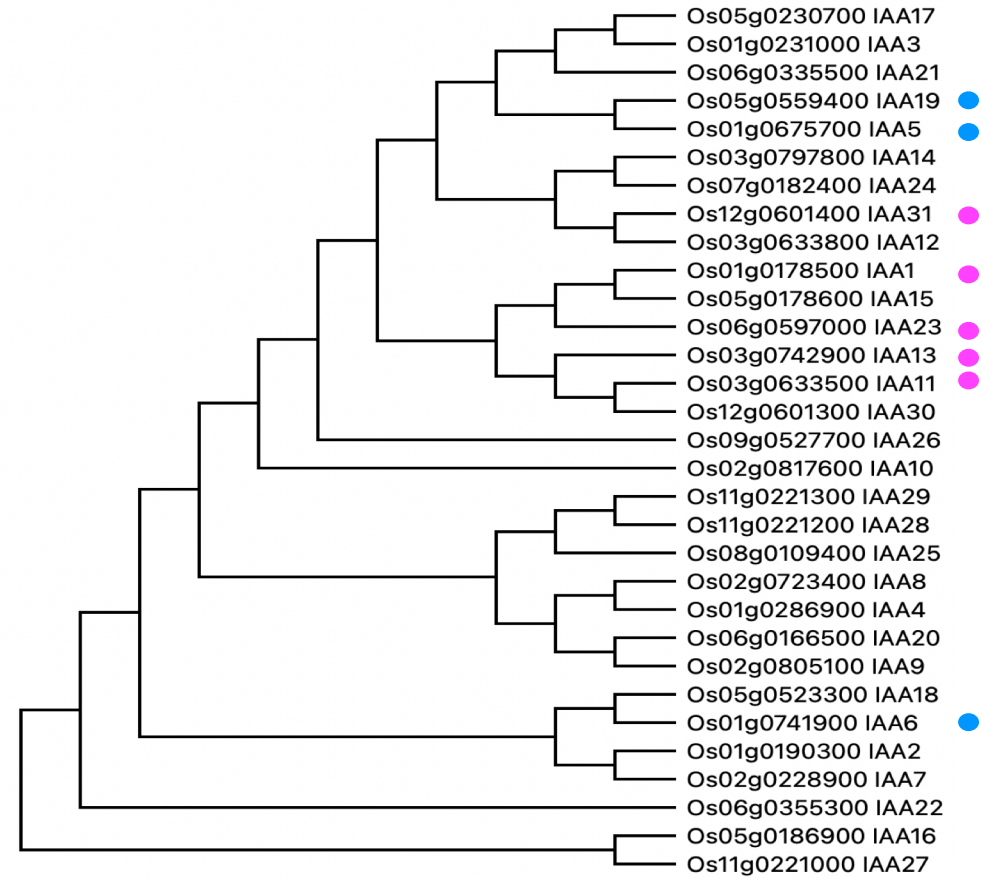

Supplement: Supplementary file 6 — Additional file 6. Fig. S5. Phylogenetic tree of the AUX/IAA family genes inferred using the Neighbor-Joining method in MEGA X software (Stecher et al. 2020). Genes previously suggested to be involved in lateral root traits are shown by magenta circles, while genes that were found in close proximity with QTL from the current study are shown by blue circles. [file 12284_2023_654_MOESM6_ESM.pdf]
