# Supplementary material for: Novel QTL for Lateral Root Density and Length Improve Phosphorus Uptake in Rice (Oryza sativa L.)
Source: Rice (N Y). 2023 Aug 24;16:37. doi: 10.1186/s12284-023-00654-z (PMC10449758; doi:10.1186/s12284-023-00654-z)
Supplement: Supplementary file 2 — Additional file 2. Table S1. Genotypic differences in lateral root traits under normal input (+P) and low input (−P) field conditions grown in Ishigaki in 2021. [file 12284_2023_654_MOESM2_ESM.docx]

**Supplementary Table S1**. Genotypic differences in lateral root traits under normal input (+P) and low input (-P) field conditions grown in Ishigaki in 2021.

| **Root trait** | **DJ123** | |  | **Nerica4** | |  | **NDJ188** | | ***Anova test of the P effect*** | | |
| --- | --- | --- | --- | --- | --- | --- | --- | --- | --- | --- | --- |
|  | ***+P*** | ***-P*** |  | ***+P*** | ***-P*** |  | ***+P*** | ***-P*** | **Average (+P)** | **Average**  **( -P)** | ***P*-value** |
| L-type root density on crown root (LDC) | 3.00 (0.50)**^a^** | 3.33 (0.29)**^a^** |  | 2.33 (0.58)**^b^** | 2.50 (0.0)**^ab^** |  | 2.67 (0.29)**^a^** | 3.00 (0.50)**^a^** | 2.67 (0.33) | 2.94 (0.41) | 0.421 |
| S-type density on crown root (SDC) | 6.67 (0.29)**^a^** | 5.0 (1.32)**^ab^** |  | 5.83 (0.29)**^a^** | 4.17 (0.76)**^a^** |  | 7.17 (1.04)**^a^** | 5.17 (0.29)**^a^** | 6.55 (0.67) | 4.78 (0.53) | 0.023**^*^** |
| S-type density on L-type  (SDL) | 11.3 (0.58)**^a^** | 12.5 (0.50)**^a^** |  | 11.5 (0.87)**^a^** | 6.83 (0.76)**^b^** |  | 11.0 (0.50)**^a^** | 9.33 (0.58)**^b^** | 11.2 (0.25) | 9.55 (2.84) | 0.355 |
| Single L-type length on crown root (LLC) | 72.8 (7.65)**^a^** | 59.8 (6.4)**^ab^** |  | 79.5 (32.7)**^a^** | 39.0 (4.21)**^b^** |  | 76.1 (7.22)**^a^** | 45.1 (12.8)**^ab^** | 76.1 (3.35) | 48.0 (10.7) | 0.012**^*^** |
| Single S-type length on crown root (SLC) | 7.23 (1.33)**^a^** | 6.37 (0.32)**^a^** |  | 7.21 (0.49)**^a^** | 6.31 (0.47)**^a^** |  | 7.91 (1.34)**^a^** | 6.48 (0.77)**^a^** | 7.45 (0.40) | 6.39 (0.81) | 0.010**^*^** |
| Single S-type length on L-type (SLL) | 6.49 (1.56)**^a^** | 4.34 (0.69)**^a^** |  | 5.68 (1.02)**^a^** | 4.43 (0.42)**^ab^** |  | 6.01 (0.35)**^a^** | 5.94 (0.67)**^a^** | 6.06 (0.40) | 4.91 (0.89) | 0.112 |

Mean phenotypic values are shown for each genotype and the number in parentheses indicates standard deviations. Different letters indicate significant differences between two P treatments within a genotype. * refers to the significant difference of the P treatments effect on lateral root traits at P<0.05 using Tukey's HSD All-Pairwise Comparisons Test
