# Supplementary material for: Novel QTL for Lateral Root Density and Length Improve Phosphorus Uptake in Rice (Oryza sativa L.)
Source: Rice (N Y). 2023 Aug 24;16:37. doi: 10.1186/s12284-023-00654-z (PMC10449758; doi:10.1186/s12284-023-00654-z)
Supplement: Supplementary file 3 — Additional file 3. Fig. S3. A linkage map containing 565 SNP markers was generated for the BC1F5 mapping population. Markers were initially ordered based on their physical map positions, but the final order was determined after applying the ripple function in Rqtl. Genetic distances were estimated in cM using the Kosambi option. [file 12284_2023_654_MOESM3_ESM.pdf]

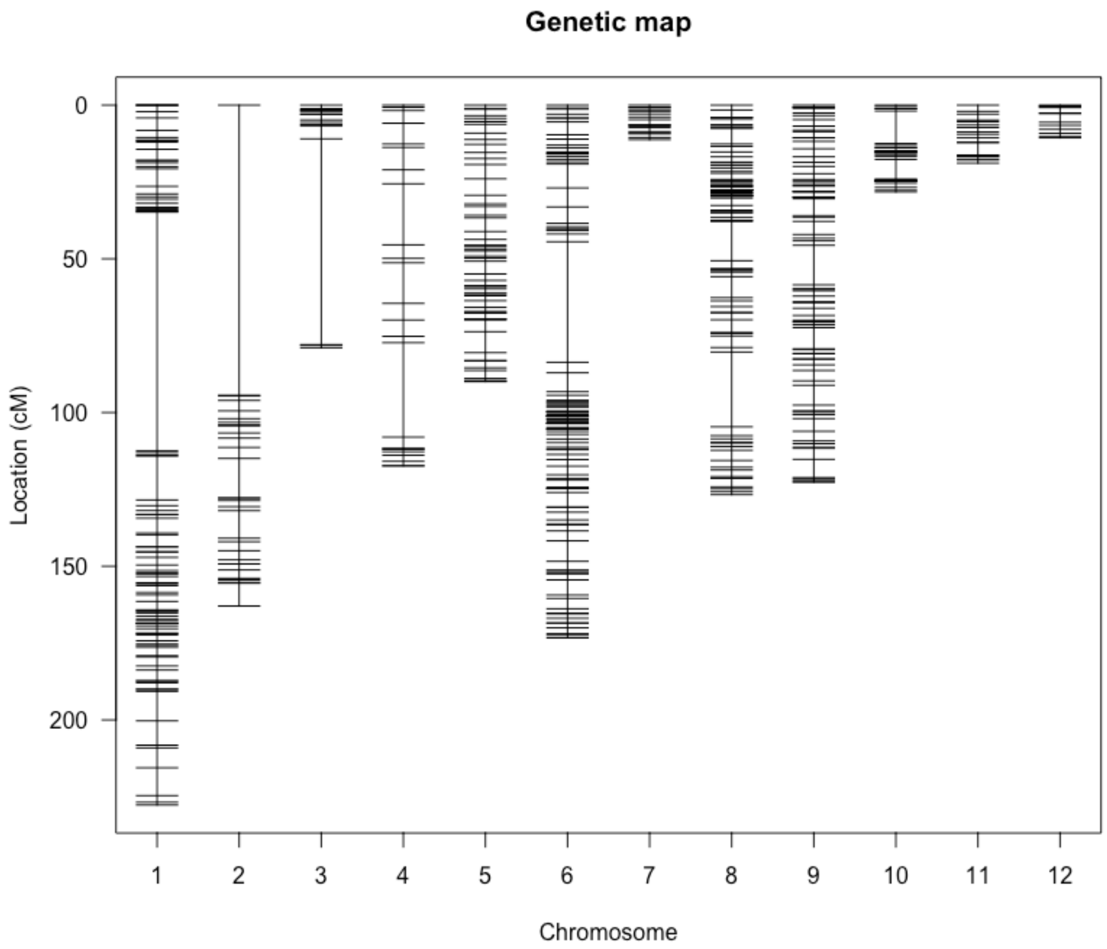

Genetic map
Location (cM)
0
50
100
150
200
Chromosome
1
2
3
4
5
6
7
8
9
10
11
12

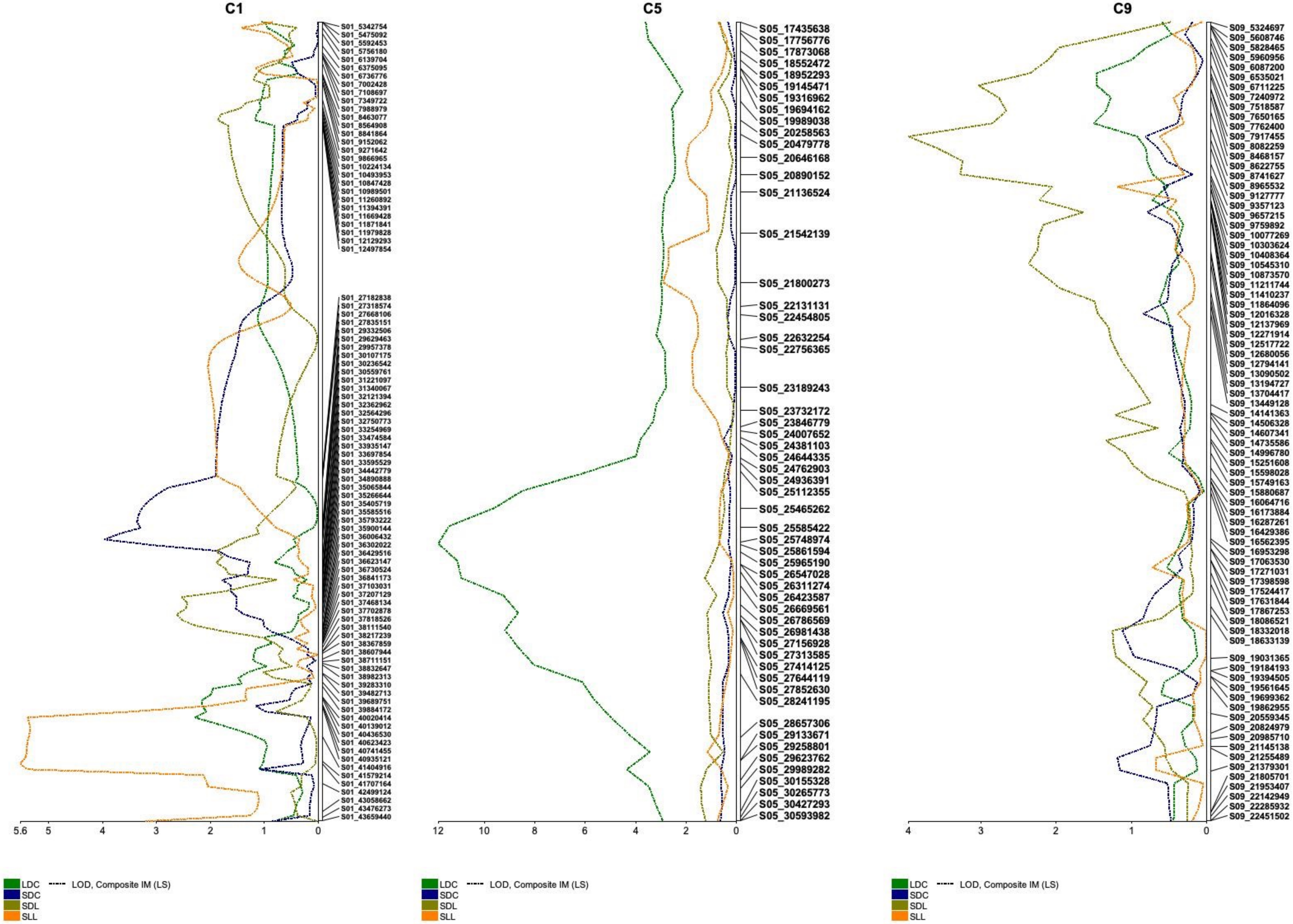
C1
S01_5342754
S01_5475092
S01_5592453
S01_5756180
S01_6139704
S01_6375095
S01_6736776
S01_7002428
S01_7108697
S01_7349722
S01_7988979
S01_8463077
S01_8564908
S01_8841864
S01_9152062
S01_9271642
S01_9866965
S01_10224134
S01_10493953
S01_10847428
S01_10989501
S01_11260892
S01_11394391
S01_11669428
S01_11871841
S01_11979828
S01_12129293
S01_12497854
S01_27182838
S01_27318574
S01_27668106
S01_27835151
S01_29332506
S01_29629463
S01_29957378
S01_30107175
S01_30236542
S01_30559761
S01_31221097
S01_31340067
S01_32121394
S01_32362962
S01_32564296
S01_32750773
S01_33254969
S01_33474584
S01_33935147
S01_33697854
S01_33595529
S01_34442779
S01_34890888
S01_35065844
S01_35266644
S01_35405719
S01_35585516
S01_35793222
S01_35900144
S01_36006432
S01_36302022
S01_36429516
S01_36623147
S01_36730524
S01_36841173
S01_37103031
S01_37207129
S01_37468134
S01_37702878
S01_37818526
S01_38111540
S01_38217239
S01_38367859
S01_38607944
S01_38711151
S01_38832647
S01_38982313
S01_39283310
S01_39482713
S01_39689751
S01_39884172
S01_40020414
S01_40139012
S01_40436530
S01_40623423
S01_40741455
S01_40935121
S01_41404916
S01_41579214
S01_41707164
S01_42499124
S01_43058662
S01_43476273
S01_43659440
5.6
5
4
3
2
1
0
LDC
SDC
SDL
SLL
LOD, Composite IM (LS)
C5
S05_17435638
S05_17756776
S05_17873068
S05_18552472
S05_18952293
S05_19145471
S05_19316962
S05_19694162
S05_19989038
S05_20258563
S05_20479778
S05_20646168
S05_20890152
S05_21136524
S05_21542139
S05_21800273
S05_22131131
S05_22454805
S05_22632254
S05_22756365
S05_23189243
S05_23732172
S05_23846779
S05_24007652
S05_24381103
S05_24644335
S05_24762903
S05_24936391
S05_25112355
S05_25465262
S05_25585422
S05_25748974
S05_25861594
S05_25965190
S05_26547028
S05_26311274
S05_26423587
S05_26669561
S05_26786569
S05_26981438
S05_27156928
S05_27313585
S05_27414125
S05_27644119
S05_27852630
S05_28241195
S05_28657306
S05_29133671
S05_29258801
S05_29623762
S05_29989282
S05_30155328
S05_30265773
S05_30427293
S05_30593982
12
10
8
6
4
2
0
LDC
SDC
SDL
SLL
LOD, Composite IM (LS)
C9
S09_5324697
S09_5608746
S09_5828465
S09_5960956
S09_6087200
S09_6535021
S09_6711225
S09_7240972
S09_7518587
S09_7650165
S09_7762400
S09_7917455
S09_8082259
S09_8468157
S09_8622755
S09_8741627
S09_8965532
S09_9127777
S09_9357123
S09_9657215
S09_9759892
S09_10077269
S09_10303624
S09_10408364
S09_10545310
S09_10873570
S09_11211744
S09_11410237
S09_11864096
S09_12016328
S09_12137969
S09_12271914
S09_12517722
S09_12680056
S09_12794141
S09_13090502
S09_13194727
S09_13704417
S09_13449128
S09_14141363
S09_14506328
S09_14607341
S09_14735586
S09_14996780
S09_15251608
S09_15598028
S09_15749163
S09_15880687
S09_16064716
S09_16173884
S09_16287261
S09_16429386
S09_16562395
S09_16953298
S09_17063530
S09_17271031
S09_17398598
S09_17524417
S09_17631844
S09_17867253
S09_18086521
S09_18332018
S09_18633139
S09_19031365
S09_19184193
S09_19394505
S09_19561645
S09_19699362
S09_19862955
S09_20559345
S09_20824979
S09_20985710
S09_21145138
S09_21255489
S09_21379301
S09_21805701
S09_21953407
S09_22142949
S09_22285932
S09_22451502
4
3
2
1
0
LDC
SDC
SDL
SLL
LOD, Composite IM (LS)

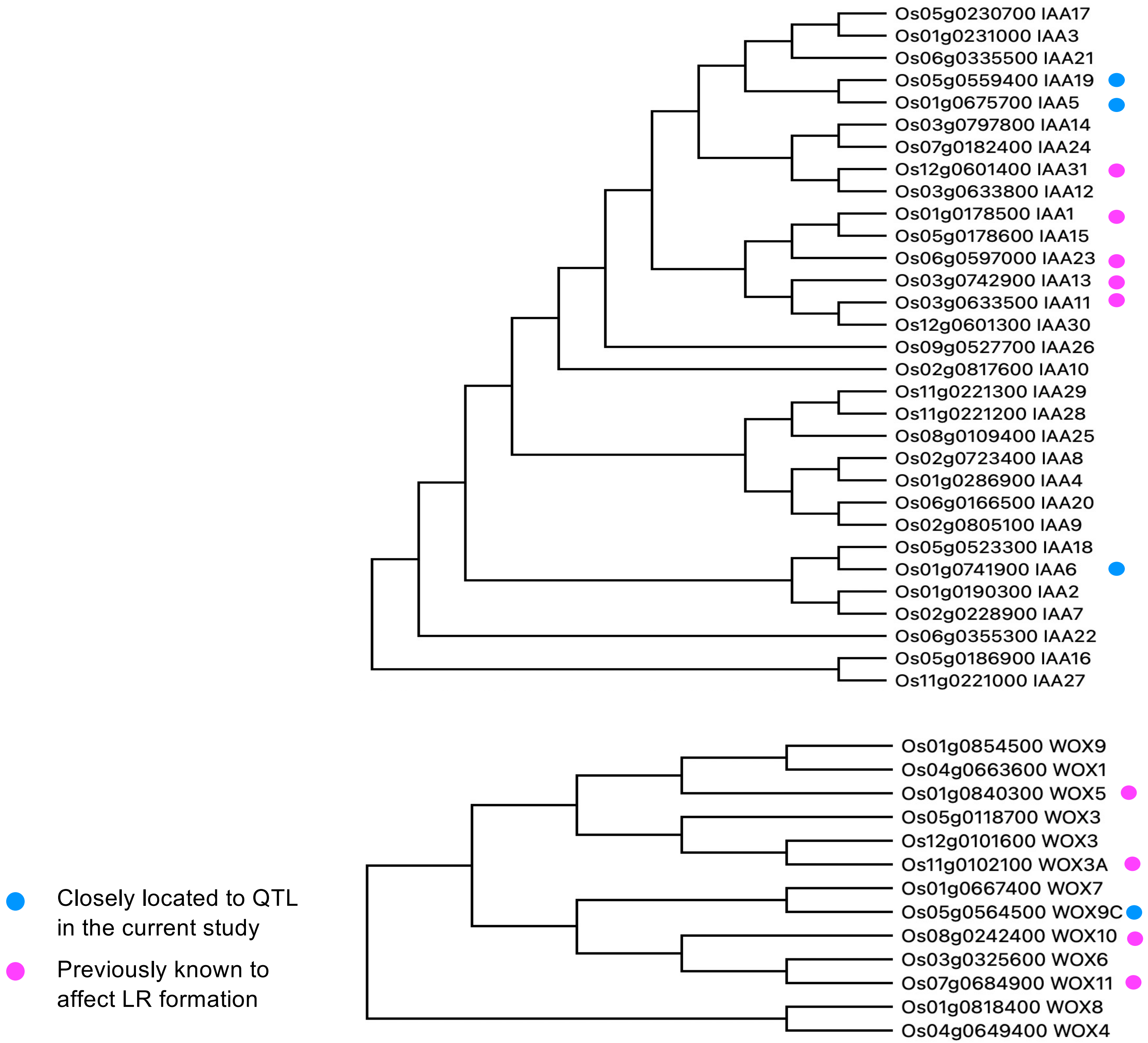
Os05g0230700 IAA17
Os01g0231000 IAA3
Os06g0335500 IAA21
Os05g0559400 IAA19
Os01g0675700 IAA5
Os03g0797800 IAA14
Os07g0182400 IAA24
Os12g0601400 IAA31
Os03g0633800 IAA12
Os01g0178500 IAA1
Os05g0178600 IAA15
Os06g0597000 IAA23
Os03g0742900 IAA13
Os03g0633500 IAA11
Os12g0601300 IAA30
Os09g0527700 IAA26
Os02g0817600 IAA10
Os11g0221300 IAA29
Os11g0221200 IAA28
Os08g0109400 IAA25
Os02g0723400 IAA8
Os01g0286900 IAA4
Os06g0166500 IAA20
Os02g0805100 IAA9
Os05g0523300 IAA18
Os01g0741900 IAA6
Os01g0190300 IAA2
Os02g0228900 IAA7
Os06g0355300 IAA22
Os05g0186900 IAA16
Os11g0221000 IAA27
Os01g0854500 WOX9
Os04g0663600 WOX1
Os01g0840300 WOX5
Os05g0118700 WOX3
Os12g0101600 WOX3
Os11g0102100 WOX3A
Os01g0667400 WOX7
Os05g0564500 WOX9C
Os08g0242400 WOX10
Os03g0325600 WOX6
Os07g0684900 WOX11
Os01g0818400 WOX8
Os04g0649400 WOX4
Closely located to QTL in the current study
Previously known to affect LR formation
